# Structure-Based Strategy Reveals a Novel Ligand Binding Site in *Staphylococcus aureus* Catabolite Control Protein A: Implications for Transcriptional Regulation and Drug Design

**DOI:** 10.1021/acsomega.6c00305

**Published:** 2026-05-14

**Authors:** André Borges Farias, Maria Carolina Sisco, Maiana de Oliveira Cerqueira e Costa, Ernesto Perez-Rueda, Marisa Fabiana Nicolás

**Affiliations:** † Laboratório Nacional de Computação Científica, Laboratório de Bioinformática, Av. Getúlio Vargas, 333, Petrópolis 25651-075, Brazil; ‡ Instituto de Investigaciones en Matemáticas Aplicadas y en Sistemas, Unidad Académica del Estado de Yucatán, Universidad Nacional Autónoma de México, Carretera Sierra Papacal—Chuburná. Km. 5.5., Mérida 97302, Mexico

## Abstract

*Staphylococcus aureus* is a major human pathogen responsible for a broad spectrum of infections and is recognized by the World Health Organization as a high-priority antimicrobial-resistant organism. The global transcription factor (TF) catabolite control protein A (CcpA), a central regulator of metabolism and virulence, represents a promising drug target. However, efforts to design inhibitors are hindered by their closed crystallographic conformation and the absence of well-defined ligand-binding cavities. Here, we present a structure-guided alignment strategy to identify potential ligand-binding sites in CcpA from *S. aureus* (Sa-CcpA), using a curated set of cocrystallized TFs as structural templates. This methodology identified a putative ligand-binding site in Sa-CcpA that was not predicted by conventional cavity-detection methods. Phylogenetic analysis showed that residues within this predicted cavity are conserved in a subset of particular pathogenic species in the order *Bacillales*, suggesting an evolutionarily constrained and functionally relevant region. To assess the stability of this newly identified site, multiple molecular dynamics simulations were performed, followed by docking analyses of two known Sa-CcpA inhibitors. Together, our results suggest that this structure-informed alignment strategy is a promising approach to uncover previously unrecognized ligand-binding regions in TFs. Although demonstrated here using Sa-CcpA as a case study, this approach may support future efforts to rationalize inhibitor design targeting transcriptional regulators.

## Graphical abstract

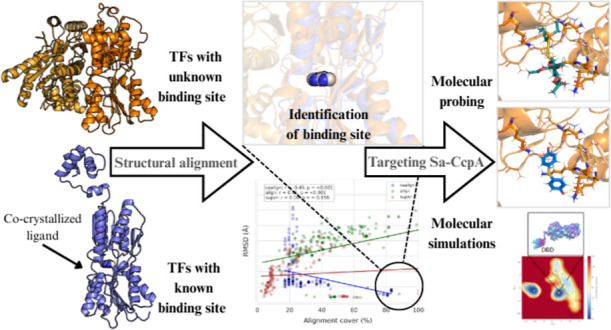

## Introduction

*Staphylococcus aureus* is a Gram-positive bacterium that asymptomatically colonizes human mucosal surfaces and moist skin regions.^1^ It is a prevalent human pathogen associated with health-care and community-acquired infections, including skin and soft tissue infections,^2^ endocarditis,^3^ osteomyelitis,^4^ bacteremia,^5,6^ and potentially lethal pneumonia.^7,8^ Among its variants, methicillin-resistant *S. aureus* (MRSA) strains represent a major global health threat due to their high resistance to beta-lactam antibiotics, aminoglycosides, macrolides, lincosamides, streptogramin B, and tetracyclines.^9,10^

A major factor contributing to antibiotic-resistant and chronic *S. aureus* infections is its ability to form biofilms,^11,12^ where the extracellular matrix acts as a protective barrier that enhances bacterial adhesion, communication mediated by quorum sensing, immune evasion, and antibiotic resistance.^12^ Biofilms limit the penetration of antimicrobials, and promote the emergence of persister cells in S. aureus, thereby increasing antibiotic tolerance.^11^ Additionally, the biofilm environment facilitates horizontal gene transfer, further contributing to antimicrobial resistance (AMR).^12^

These biofilm-associated mechanisms not only exacerbate AMR but also limit the efficacy of conventional antibiotics, highlighting a significant gap in current antimicrobial drug discovery efforts. Several factors contribute to this crisis, including high costs, long development times, and the rapid emergence of resistance. Contributing factors include the scarcity of compounds with novel mechanisms of action, the limited discovery of small molecules that could be membrane-permeable and selective, and the ongoing difficulty in identifying effective molecular targets.^13–16^ Together, these challenges highlight the need for alternative therapeutic strategies that go beyond conventional targets.

Recent advances in computational biology have opened new avenues for antimicrobial discovery, particularly through target identification, structure-based drug design, and machine learning-based compound screening.^17–20^ These tools have allowed the discovery of novel antibiotics and peptide-based therapeutics with activity against resistant strains.^21–23^ Furthermore, molecular dynamics techniques have also been shown to be promising for investigating protein conformational dynamics,^24–26^ as well as characterization of the interaction profiles of compounds within the binding sites. In this context, transcription factors (TFs) have emerged as promising targets due to their central role in the regulation of virulence, metabolism, and stress response related-genes in bacteria.^27,28^ However, there is still a lack of deep structural data information, particularly on their ligand binding sites.^29^

Among *S. aureus* TFs, the Catabolite Control Protein A (CcpA), a highly conserved member of the LacI/GalR family, controls carbon catabolite responses that help microbes rapidly adapt to environmental changes.^30^ In *S. aureus* and other Gram-positive pathogens, CcpA also plays pleiotropic roles in biofilm formation, antibiotic resistance, and adaptation to tissue-specific host environments.^31–35^ Furthermore, CcpA has been described as a global regulator in the transcriptional regulatory network (TRN) of *S. aureus* Bmb9393 (ST239), with a prominent role in controlling the expression of biofilm-associated genes.^36^

In this context, the most well-characterized CcpA protein is that of *Bacillus subtilis* (Bs-CcpA), which is composed of an N-terminal DNA-binding domain (DBD residues 1–59), and a C-terminal dimerization domain (residues 60–334) and binds to more than 50 operator sequences known as catabolite response elements (*cre*). Its regulatory function is modulated through the interaction between the C-terminal dimerization domain and the phosphoprotein HPr, which is phosphorylated in Ser46 by its cognate kinase HPrK in the presence of glucose. When CcpA binds to HPr phosphorylated, the CcpAHPr-Ser46-P complex undergoes conformational changes that stabilize its dimeric structure for DNA binding, thus increasing its affinity for *cre* sites and defining its regulatory role.^37^

Although recent studies^38,39^ have identified small-molecule inhibitors capable of disrupting the function of Sa-CcpA, the mechanistic details of their binding remain only partially understood. Two notable compounds have emerged from the literature: 4,4-thiodiphenol (TDP) and the gold-based drug auranofin.

TDP was identified through a thermal shift-based screening followed by rigid docking simulations, which suggested that its binding occurs at the interdomain interface between the periplasmic binding protein (PBP)-like domain and the DNA-binding domain (DBD). Site-directed mutagenesis indicated a possible role for Leu55 in TDP interaction, although direct binding to this residue has not been conclusively demonstrated.^39^ Auranofin, on the other hand, was proposed to bind directly to the dimerization domain through the two conserved cysteine residues, Cys216 and Cys242. As reported by Lin et al. (2025), mutation of these residues to serine abolishes the thermal shift response and significantly reduces the inhibitory activity of auranofin, suggesting that intact cysteines are required for its action.^38^ However, Liao et al. (2022) demonstrated that oxidation of Cys216 and Cys242 by Cu­(II) induces tetramerization and disrupts DNA binding, indicating that perturbation of these residues can trigger large-scale conformational changes, including disruption of the functional dimeric conformation of CcpA and impairment of its regulatory activity.^40^ Therefore, mutation of both cysteines may alter the global conformational landscape of CcpA. These findings are primarily based on mutagenesis and thermal shift assays and lack direct structural evidence confirming that these cysteine residues are part of the ligand-binding site. Notably, these residues are located within the dimerization domain, and their mutations disrupt the oligomeric conformation of CcpA and its DNA-binding capacity. Consequently, diverse aspects of the structural dynamics and conformation of the binding site and the DBD remain unclear. To address these limitations, we employed a bioinformatic approach that combines structural alignment of Sa-CcpA against a collection of TFs cocrystallized with ligands to identify potential druggable cavities. Subsequently, molecular dynamics simulations were performed to evaluate the stability of the predicted cavities, followed by molecular docking analyses with TDP and auranofin to explore potential binding modes and support the rational design of Sa-CcpA inhibitors.

## Results and Discussion

### The Plasticity of DBD Motif from Sa-CcpA Structure

Initially, we obtained a crystallographic structure of CcpA from the PDB Data Bank (PDB 7E5W)^40^ of *S. aureus*, strain N315. Sa-CcpA closely resembles that of *Bacillus subtilis* CcpA (Bs-CcpA, PDB 1ZVV)^40,41^ and *E. coli* PurR (Ec-PurR, PDB 1BDH)^42^ with a root-mean-square deviation (RMSD) of 1.70 Å and 1.98 Å for 239 and 222 Cα atoms, respectively (Figure 1A). However, a notable difference is observed in the DNA-binding domain, where two distinct orientations can be identified (Figure 1B). In Bs-CcpA, the four helices of the DNA-binding domain are presented in an extended conformation, while the hinge helix of the Sa-CcpA DBD (residues 50–57) appears disordered, providing conformational flexibility for interactions at the dimeric interface^40^ (Figure 1C,D).

**Figure 1:**
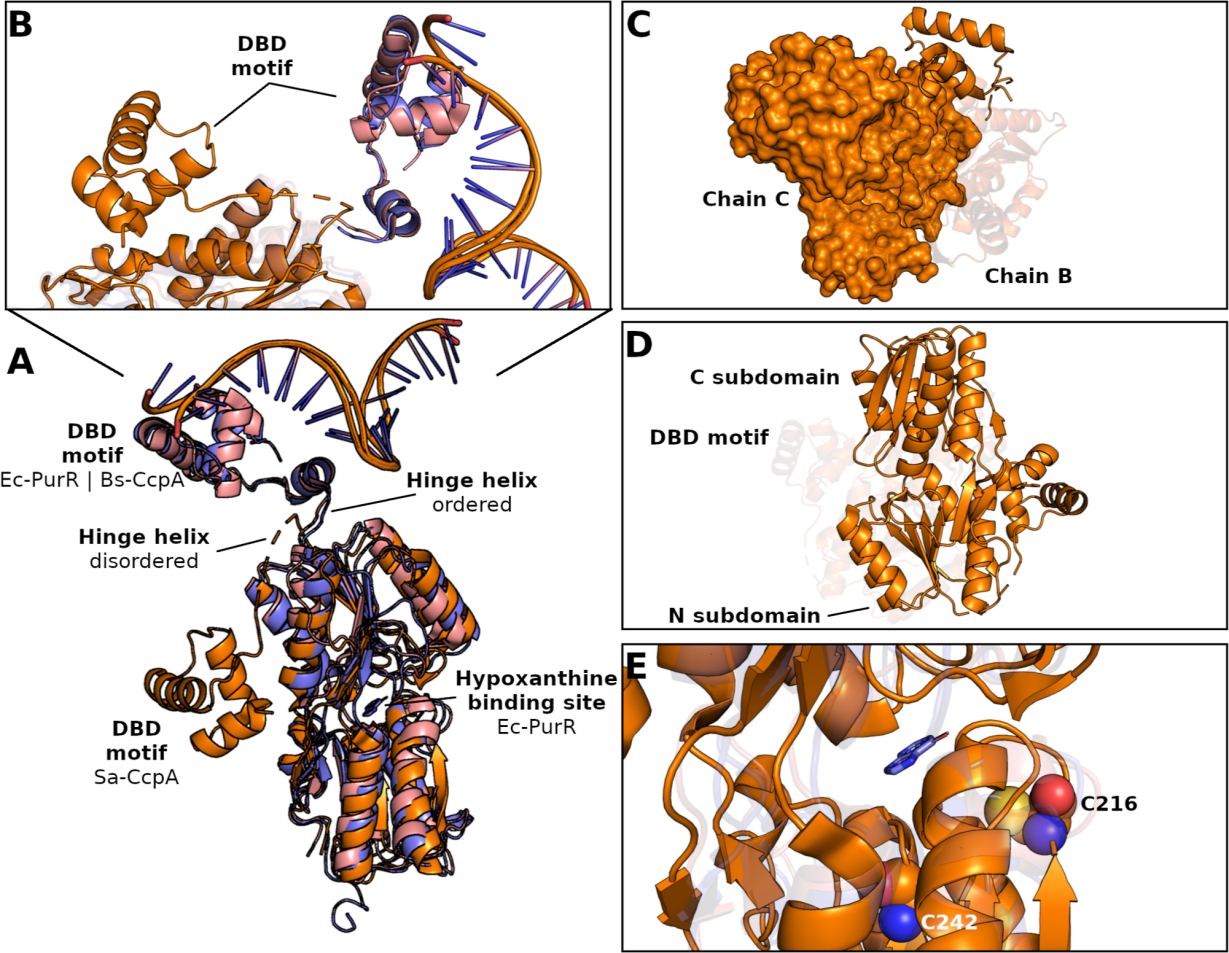
Overview of CcpA structure. Structural alignment of *S. aureus* CcpA (Sa-CcpA, PDB: 7E5W), *B. subtilis* CcpA (Bs-CcpA, PDB: 1ZVV), and *E. coli* PurR (Ec-PurR, PDB: 1BDH) (A), highlighting the conformational changes in the DBD motif (B). The Sa-CcpA structure adopts a dimeric organization (C; chains C and B), illustrating the N-terminal and C-terminal subdomains (D). Structural superposition of Ec-PurR with Sa-CcpA revealed that the hypoxanthine-binding site in Ec-PurR may also serve as a potential binding site in Sa-CcpA, highlighting two cysteine residues (Cys216 and Cys242) that are critical for dimerization (E). Sa-CcpA, Bs-CcpA, and Ec-PurR are shown in orange, pink, and blue, respectively.

To further investigate this disordered region and better assess its impact on protein stability and potential conformational changes, we applied comparative modeling to reconstruct the hinge helix. The resulting model exhibited over 90% of amino acids in favored regions (Figure S1). In addition, we performed molecular dynamics simulations on both the monomeric and dimeric forms of the protein in three distinct conformations: (i) crystallographic conformation, where the DNA-binding domain (DBD) is oriented toward the dimeric interface; (ii) extended conformation, where the DBD adopts an open orientation similar to Bs-CcpA; and (iii) the DNA-bound conformation, representing the complex between CcpA and its Transcription Factor Binding Site (TFBS).

The RMSD (Root Mean Square Deviation) plots (Figure S2A–F) present the comparison of stability of these conformations and provide a measure of structural deviation over time relative to the initial conformation, reflecting the overall stability and conformational changes of the system during simulation, revealing different dynamic behaviors across the distinct configurations. The CcpA monomer in the extended conformation exhibited a significantly higher RMSD (≈1.2 nm) than the DNA-bound and crystallographic forms, suggesting greater structural deviation over the simulation period. Similarly, the dimer in the extended conformation also displayed higher RMSD values than its dimer in the crystallographic forms, suggesting that interactions between the DBD and the chain promote greater molecular stability. The Root Mean Square Fluctuation (RMSF) analysis (Figure S3A–F) further highlights these differences by measuring residue-level flexibility. In the monomeric state, especially in the extended and DNA-bound conformation, higher fluctuations were observed in the DBD, particularly within residues 50–57, corresponding to the hinge helix previously described as disordered.^40^ This observation suggests a degree of conformational plasticity in this region, which could influence DNA-binding interactions. Conversely, the dimeric configurations exhibited lower residue fluctuations (Figure S3A–F), indicating a more stable structural organization. These findings support the hypothesis that the DBD, particularly the hinge helix, exhibits conformational flexibility, which was more pronounced in the monomeric state in extended and DNA bounded simulations. Such flexibility could be relevant for the transcription factor’s functional mechanisms and may be crucial for its interaction with the TFBS.

Principal component analysis (PCA) revealed the most relevant motions of Sa-CcpA in both monomeric and dimeric forms. For this analysis, the last 100 ns of each trajectory of the three replicas were concatenated, totaling 300 ns, and the covariance matrix was calculated based on the Cα atoms. The diagonalization of this matrix yielded eigenvectors and eigenvalues, representing the directions of motion and their corresponding amplitudes, respectively. The PCA results (Figures S7 and S8) highlighted large amplitude motions of the DBD motif, particularly in the monomeric form of Sa-CcpA, both bound to DNA and in the extended conformation (Figure S7B,C).

Once the most relevant movements along the trajectory were identified, we selected the two most relevant principal components (PC1 and PC2) as collective variables to calculate the free energy landscape (FEL). We can observe two different conformational dynamics for the monomeric form of Sa-CcpA (Figure 2A,B), in which a predominant stable conformation is observed for the crystallization state. On the other hand, when observing the energy profile of the monomer simulated with DNA, we observed multiple shallow basins, suggesting a higher conformational flexibility of DBD (Figure 2B). In addition, the low energy barrier observed in replica 1, suggests an easy transition between conformational states, potentially reflecting functional dynamics for this transcription factor. Similarly, the conformational landscape of the dimer showed the conformational flexibility of DBD more pronounced in the DNA-bounded system (Figure 2D) compared to the crystallization state (Figure 2C). This finding suggests that the plasticity of DBD is necessary for the correct interaction with its binding site in the DNA.^43,44^ Of note, the energy profile of the main motion, revealing the mechanism that enhances DNA target recognition, highlights the flexibility of the motif to conformation, stabilizing into a well-defined state required for regulatory activity.

**Figure 2:**
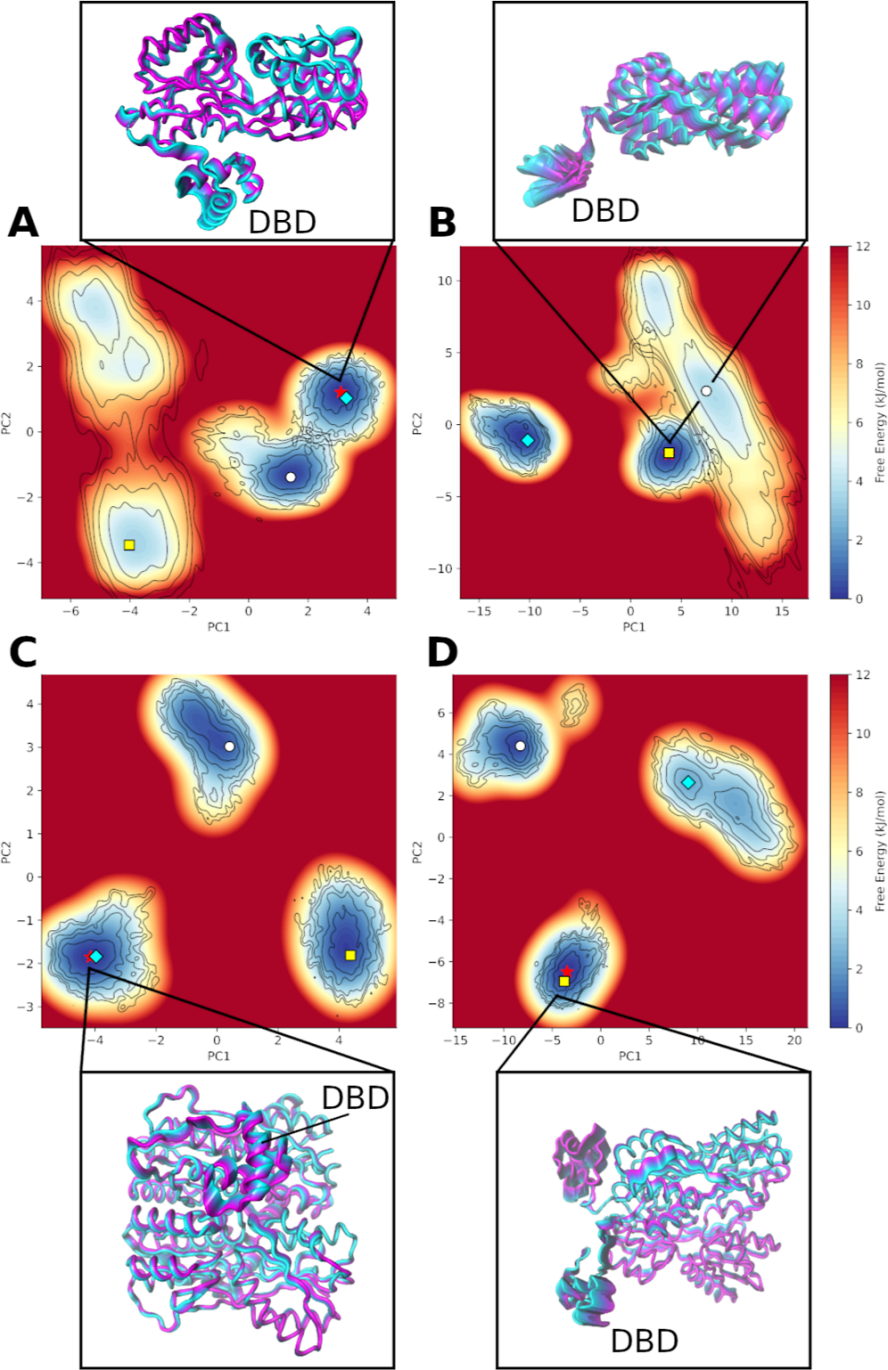
The free energy landscape of the main motion of Sa-CcpA. The crystallographic and DNA-bounded conformation of monomer (A,B) and dimer (C,D), respectively. The symbols denote the minimum energy of each replica: the star denotes the global minimum, and the circle, diamond, and square denote replicas 1, 2, and 3, respectively. We illustrate the motion of DBD in the replica which present the global energy.

### Identification and Characterization of Potential Binding Sites in Sa-CcpA for Structure-Based Drug Design

Following the structural analysis of Sa-CcpA, we identified potential binding sites for the design of new inhibitors. To achieve this, we used CavityPlus^45^ to search in the crystallographic structure for suitable cavities for the small-molecule binding site (Figures S4). Although cavity searching is a widely used approach for identifying potential binding sites in drug design, its effectiveness is highly dependent on the protein structural conformation. For Bs-CcpA and Ec-PurR, where the binding cavities were accessible, the algorithm successfully detected the pockets. However, for Sa-CcpA, the results were not as promising and point to the fact that the search for binding cavities based on structural topology is not always an effective strategy because it relies heavily on the crystallographic structure. Table S1 summarizes the predicted cavities for Sa-CcpA, Bs-CcpA, and Ec-PurR. In particular, the only cavity predicted with medium druggability in Sa-CcpA was located within the DNA-binding domain, which corresponds to a completely different region compared to the homologous proteins Bs-CcpA and Ec-PurR.

The conformation observed during crystallization may represent a slightly closed state, which can hinder the identification of significant binding sites. To evaluate the openness of the cavity, we used an analysis of the Solvent Accessible Surface Area (SASA) of the predicted cavity throughout the MD trajectory (Figure 3).

**Figure 3:**
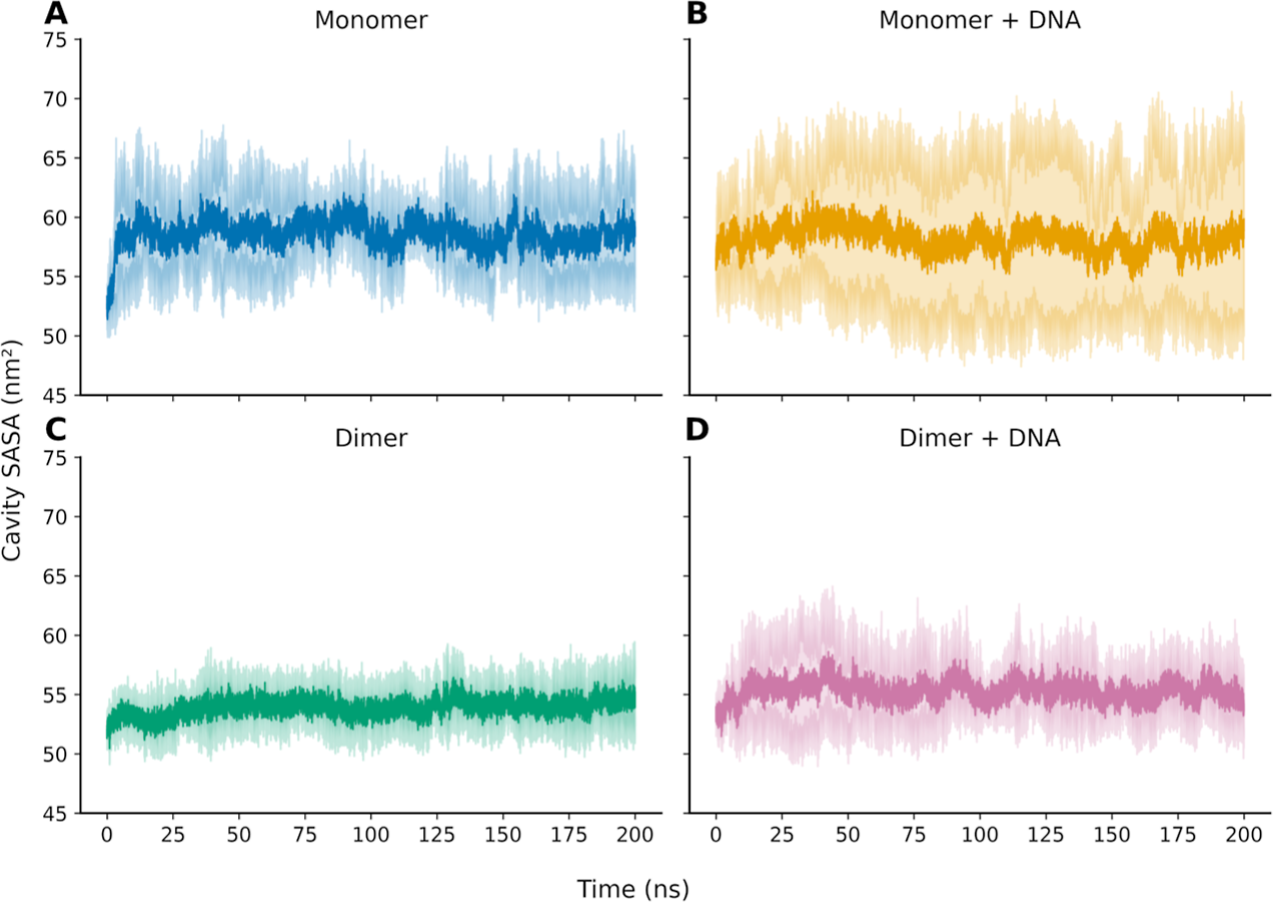
Solvent accessible surface area of the predicted cavity throughout molecular dynamics simulations. The plots show the mean SASA values (bold line) with the corresponding standard deviation calculated from the trajectories (pastel color). (A,B) Correspond to the monomeric systems starting from the crystallographic conformation and from the DNA-bound conformation, respectively. (C,D) Show the corresponding analyses for the dimeric systems starting from the crystallographic and DNA-bound conformations.

For the monomeric system (Figure 3A), the cavity initially appears relatively closed, with SASA values around 50 nm^2^. However, an early increase in solvent exposure is observed within the first ≈25 ns, indicating a rapid opening of the cavity. Subsequent fluctuations in SASA are observed across the trajectories, with values typically ranging between ≈55 and ≈65 nm^2^. In the monomer bound to DNA (Figure 3B), transient opening events are also observed, but occur at different time intervals. For example, replica 1 shows a marked increase in SASA between approximately 25 and 50 ns (from ≈52 to ≈65 nm^2^), while replica 3 exhibits a later opening event between ≈130 and 180 ns, reaching values up to ≈70 nm^2^ (see Figure S10).

For the dimeric system (Figure 3C), the cavity again appears initially less exposed (≈50 nm^2^), followed by an increase in SASA during the first ≈25 ns. In this system, although fluctuations are present, they are comparatively less pronounced than those observed for the monomeric systems. Finally, in the dimer bound to DNA (Figure 3D), all replicas display transitions between less solvent-exposed and more solvent-exposed states throughout the simulations, indicating dynamic fluctuations in cavity accessibility. These results indicate that the predicted cavity undergoes reproducible transitions between more closed and more solvent-exposed conformations across independent simulations, supporting the hypothesis that the pocket is transiently formed as a consequence of the intrinsic conformational plasticity of the protein.

Taking into account this limitation, we consider that a structure-based approach in homology can be a promising strategy for discovering potential binding sites. In this way, we used the EntraF database to construct a data set of *E. coli* TFs with cocrystallized ligands in their structures. Importantly, although the current TF structural space is still limited to Gram-positive protein, studies analyzing sequence–structure similarity relationships have shown that proteins can exhibit substantial structural similarity despite low sequence identity.^46^ This principle supports the use of structural alignment methods to identify homologous architectures even across distantly related organisms.

Subsequently, we developed a Python script using the functions available in PyMOL software to systematically align the Sa-CcpA structure against the previously curated data set of transcription factors. To ensure a comprehensive and systematic structural comparison, three alignment methods, (i) align, (ii) super, and (iii) cealign, were employed. Although all three methods aim to optimize structural superposition, they differ substantially in their underlying algorithms and sensitivity to sequence similarity align performs a sequence-dependent alignment, in which the structural fitting is guided by residue correspondences derived from sequence similarity. This approach is efficient and accurate for proteins that share moderate to high sequence identity (>30%), but it can lose accuracy when sequence conservation is low. In contrast, the super method applies a sequence-independent structural alignment based on dynamic programming, followed by iterative refinement to minimize deviations and exclude mismatched pairs. This makes super more robust for proteins with limited sequence similarity while maintaining computational efficiency. Finally, cealign employs the Combinatorial Extension (CE) algorithm, which aligns proteins exclusively on the basis of geometric features and topological correspondence, without relying on sequence information. Although computationally more demanding, cealign provides highly reliable alignments even for distantly related or structurally divergent proteins. Figure 4 shows the result of structural alignment using these three methods.

**Figure 4:**
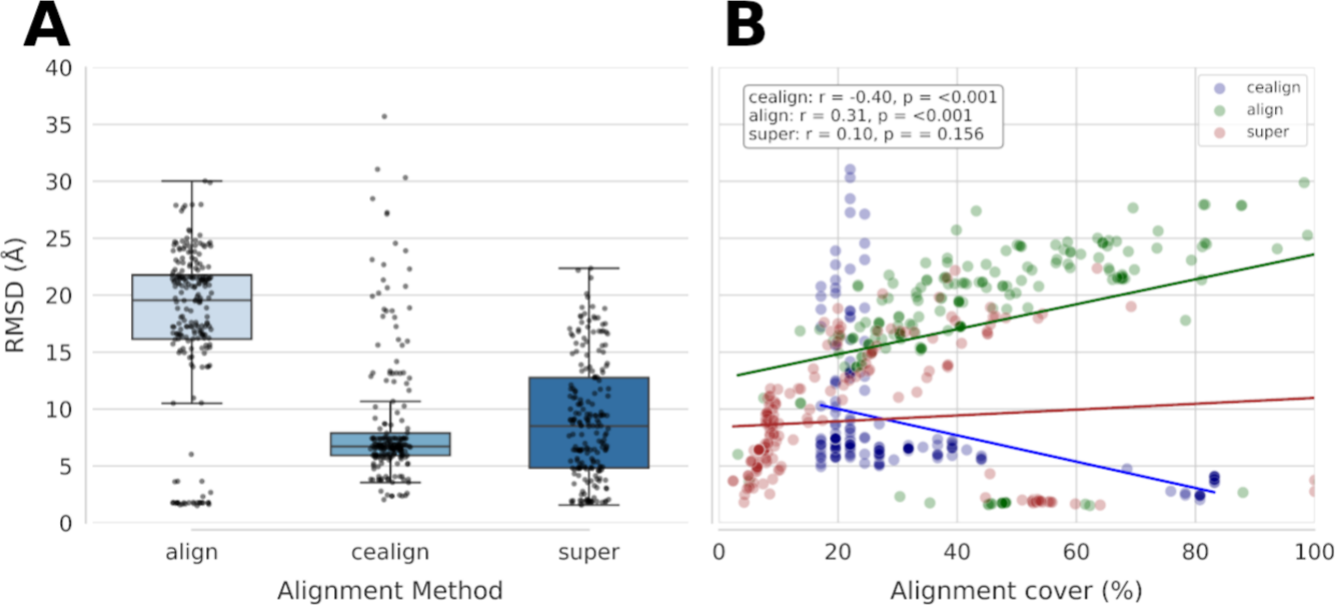
Structural alignment of Sa-CcpA against transcription factors with cocrystallized ligands obtained from the EntraF database. (A) Root mean square deviation (RMSD) values of Sa-CcpA calculated using the align, cealign, and super methods. (B) Pearson correlation between RMSD and alignment coverage using the align (green), cealign (blue), and super (brown) approaches.

Obtaining a low RMSD value with low alignment coverage is not particularly meaningful, as it may result from aligning only a few structural motifs that do not represent a relevant binding region. Therefore, low RMSD values associated with coverage greater than 60% are more informative for identifying structurally conserved regions and potential binding cavities. As shown in Figure 4, the cealign method exhibited a moderate, significant, and negative correlation between RMSD and alignment coverage, indicating that greater coverage is associated with lower RMSD values. In contrast, the align method showed a positive correlation, suggesting that broader alignments tend to increase structural deviation. Overall, the cealign method achieved lower average RMSD values than align and, importantly, identified several proteins with alignment coverage above 70% and low RMSD values (Table 1, see full results in Table S2). However, the super method, did not produce satisfactory results for this data set.

**Table 1:** Structural Alignment Obtained With Cealign for Proteins Exhibiting Both Sequence Coverage Greater than 70%

PDB aligned	Alignment cover	RMSD value (Å)
1EFA	83.180	3.546
2PE5	83.180	3.561
1HJ9	83.180	3.740
1JFS	83.180	3.792
1QP7	83.180	3.795
1QQB	83.180	3.796
1QP4	83.180	3.800
1BDH	83.180	3.827
1VPW	83.180	3.827
1JF7	83.180	3.830
1QQA	83.180	3.862
1QP0	83.180	3.878
1QPZ	83.180	4.063
1BDI	83.180	4.069
1PNR	83.180	4.074
1WET	83.180	4.074
1ZAY	83.180	4.076
1SXG	80.734	2.039
3EDC	80.734	2.346
2NZU	80.734	2.352
1LBH	80.734	2.353
2PAF	80.734	2.407
4RZT	80.734	2.444
2P9H	78.287	2.441
3TB6	78.287	2.601
4XXH	75.841	2.784

Although our structural alignment strategy is based on TFs structures from *E. coli*, it is important to emphasize that we employed three different alignment algorithms, among which cealign is particularly robust for proteins with low or no sequence similarity. This choice helps to mitigate potential bias arising from low primary sequence similarity between Gram-positive and Gram-negative TFs. From a structural biology perspective, the resulting alignments exhibited low root-mean-square deviation (RMSD) values (below 4 Å) with more than 80% structural overlapping, indicating a high degree of structural conservation despite differences in organismal origin. These results support the well-established observation that protein structures are generally more conserved than their amino acid sequences. Consequently, proteins with low sequence identity can still retain highly similar three-dimensional folds and structural motifs. This result highlights the generalization capability of the proposed strategy.

Following the structural alignment, a potential binding site was identified in a highly conserved region among homologous structures of *E. coli* PurR (PDB IDs: 1BDH, 1JFS, 1QP4, 1QP7, and 1QQB) and *E. coli* LacI (PDB IDs: 1EFA and 2PE5). Figure 5A shows the location of the predicted cavity, while Figure 5B–E illustrate the structural superpositions of Sa-CcpA with 1EFA (B), 2PE5 (C), 1BDH (D), and the overlap of the eight best alignments (E).

**Figure 5:**
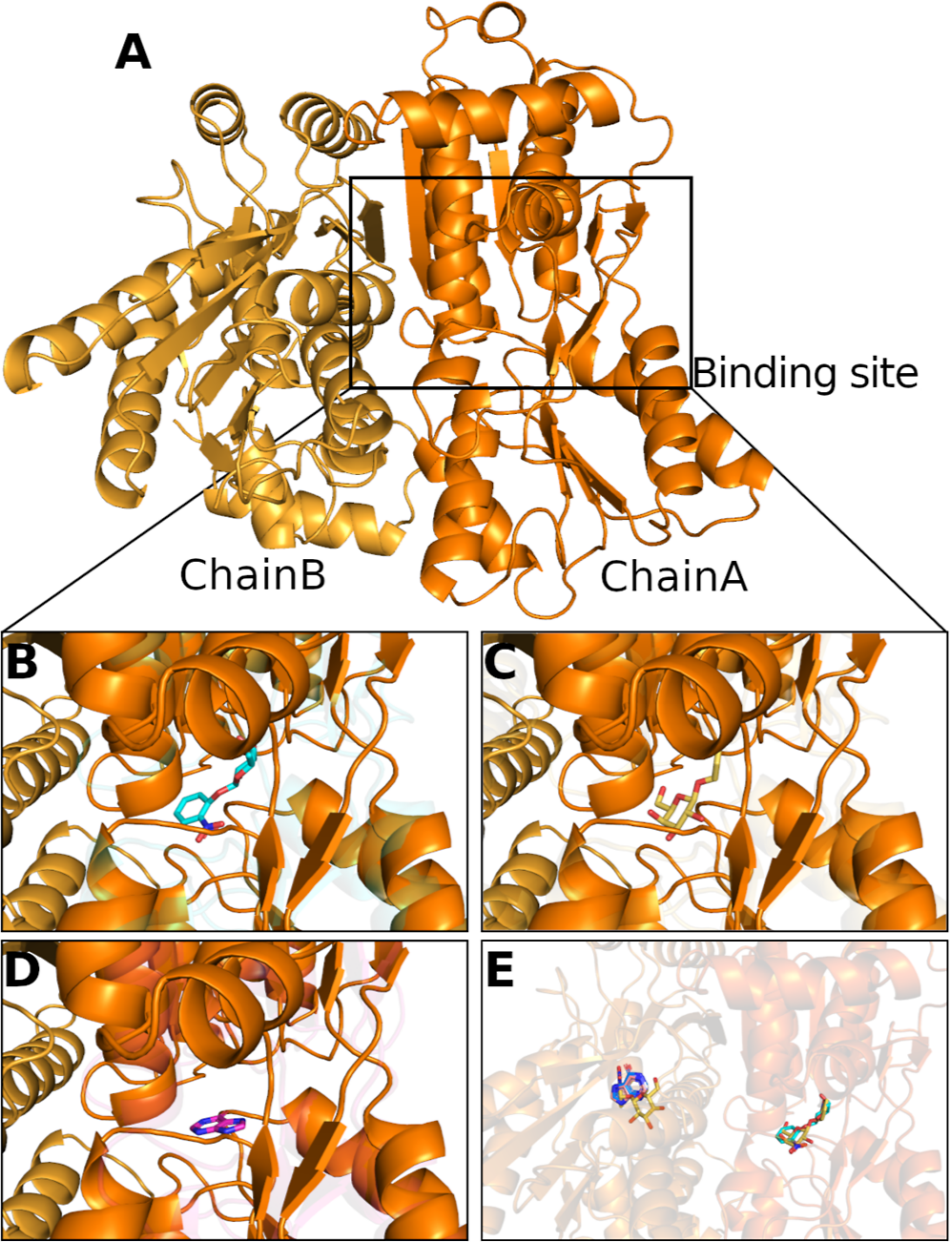
Ligand binding site predicted by structural alignment. (A) Overview of the Sa-CcpA region identified as a potential ligand-binding site. Superimposition of the Sa-CcpA structure (orange) with homologous crystallographic structures (transparent) from the Protein Data Bank: 1EFA (cyan) (B), 2PE5 (yellow) (C), and 1BDH (pink) (D). This comparison highlights a conserved cavity maintained across multiple homologues (E). Hydrogen atoms were omitted for clarity.

The spatial coincidence between these cavities suggests that Sa-CcpA may share a similar ligand-binding mode with its homologous TFs, particularly *E. coli* PurR and LacI. In both of these proteins, ligand binding occurs within an allosteric pocket located at the interface between the regulatory and DNA-binding domains, inducing conformational changes that modulate DNA affinity. Identifying a comparable cavity in Sa-CcpA implies the potential conservation of this regulatory mechanism within the LacI/GalR family.^47^ This conservation is consistent with previous structural studies showing that, despite variations in sequence identity, members of this family preserve the core topology required for allosteric regulation through effector binding. This observation suggests that ligand binding at this conserved site could influence the conformational dynamics of Sa-CcpA, ultimately affecting its interaction with TFBS. Together, these findings suggest that Sa-CcpA retains the functional architecture necessary for metabolite-dependent regulation, offering a promising starting point for future docking and molecular dynamics studies to discover potential inhibitors.

### Phylogenetic Conservation of Sa-CcpA Homologues

The structural conservation observed at the predicted ligand-binding site suggests that this region may be under evolutionary constraint. To investigate this possibility, we extended our analysis to examine the evolutionary relationships of CcpA across members of the *Bacillales* order. This approach allowed us to assess whether the structural conservation observed in Sa-CcpA reflects broader evolutionary conservation within the family. To this end, we performed a BLASTp search using the Sa-CcpA sequence as a query against *Bacillales* proteomes, followed by phylogenetic reconstruction to evaluate the conservation and divergence of CcpA homologues across related species (Figure 6A).

**Figure 6:**
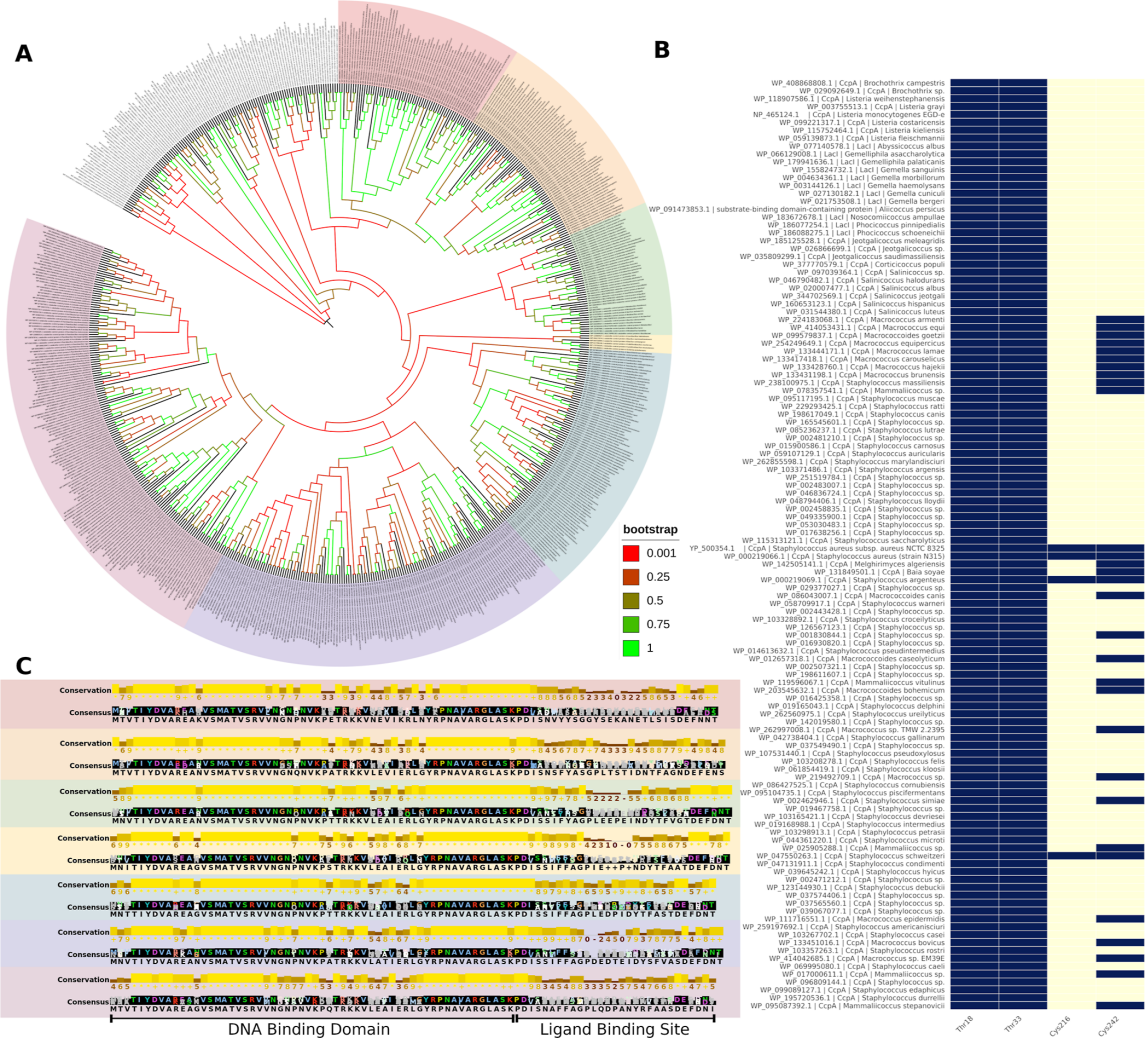
Comparative evolutionary and sequence conservation analysis of Sa-CcpA homologues. Phylogenetic tree constructed from 659 amino acid sequences showing the evolutionary relationships among CcpA homologues (A). Heatmap depicting the conservation of four key residues (Thr18, Thr33, Cys216, and Cys242); blue indicates the presence and yellow the absence of the corresponding residue in each organism. In addition to the sequences included in the phylogenetic analysis, this heatmap also incorporates homologues obtained from the nonredundant filtering performed without CD-HIT (B). Sequence logo representation by clade, illustrating the conservation pattern within the DNA-binding domain (DBD) and ligand-binding domain (LBD) (C).

We observed that clade 1 groups the most common known pathogens, such as *Listeria monocytogenes*, *S. aureus* subs. *aureus*, *Staphylococcus massiliensis* and *S. auricularis*, and some less known opportunistic pathogens as *Gemmella*, *Nosocomiicoccus*, *Macrococcus*, *Macrococcoides* and *Mammaliicoccus*. On the other hand, clade three comprises mainly nonpathogenic species, with only two reports: *Cytobacillus* isolated from human blood culture and *Metabacillus* from a laboratory animal skin infection.^48,49^ Analyzing the amino acid sequences of both clades, we observed that the hinge helix (residues 50–57 of Sa-CcpA) is highly conserved among the species of both clades, however, the dimerization domain (residues 60–329 of Sa-CcpA) is less conserved. We also observed that the only sequences with an alanine on residue 31 (inside the DBD) belong to *S. aureus*. Residue 232 is a methionine only in *S. aureus*, in the other species of both clades it can change to Leu, Iso, Phe, or Val. Residue 247 is a Glutamic acid in *S. aureus*, while it can change to Met, Gln, Val, and Leu in other species.

Currently, two distinct mechanisms for inhibition of*S. aureus* CcpA (Sa-CcpA) DNA binding has been described, each with therapeutic implications: one mediated by redox-sensitive cysteine residues within the dimerization domain, and the other involving phosphorylation of threonine residues in the DBD. Both modifications prevent Sa-CcpA’s from recognizing *cre* sites, ultimately altering the transcriptional regulation of its target genes involved in metabolism and pleiotropic functions.

In the redox-regulatory mechanism in Sa-CcpA, oxidation of two cysteine residues (Cys216 and Cys242) in the dimerization domain by copper­(II) promotes tetramerization and disrupts DNA binding.^40^ This conformational inactivation impairs the regulation of genes and represents a mechanism absent in nonpathogenic species such as *B. subtilis*. These findings suggest that targeting the dimerization domain of Sa-CcpA may compromise the adaptation of *S. aureus* to host-induced stress, highlighting the redox-regulatory mechanism as a promising therapeutic target.

In the serine/threonine kinase Stk1 phosphorylation, two threonine residues (Thr18 and Thr33) within the DBD of Sa-CcpA are critical.^50^ This modification disrupts its DNA-binding conformation and prevents transcriptional control of target genes. Structurally, this phosphorylation shifts the equilibrium toward an inactive, phosphorylated isoform of Sa-CcpA, which correlates with impaired biofilm formation. Although it remains unclear how this phosphorylation event influences the expression of the CcpA regulon, or whether it might be reversed by a serine/threonine phosphatase such as Stp, which is cotranscribed with Stk1,^51^ these findings suggest that modulating CcpA activity through phosphorylation, or alternatively activating the Stk1 kinase, could influence biofilm formation in *S. aureus* and provide a promising avenue for drug development.

As the two cysteine residues have previously been associated with the ability of *S. aureus* to resist host-mediated oxidative stress,^40^ the hypothesis of cysteine conservation in pathogenic species could be raised. Some of the species mentioned above are opportunistic pathogens in both humans and animals,^52–61^ with *S. aureus* and *Staphylococcus epidermidis* commonly associated with human nosocomial infections.^62^ However, other pathogenic species presented in the same class (*Bacilli*, belonging to the *Bacillota phylum*) such as*Listeria monocitogenes*, do not present conservation for these cysteine residues, demonstrating that this conservation feature is only present in some species. Regarding the two threonine residues mentioned above, we observed that these are strictly preserved in all species of the*Bacillales* order (Figure 6B).

Regarding the ligand binding site (Figure 6C), we observed that residues Pro68 and Asp69 are highly conserved among all clades. The next residue, Ile70, can be substituted for valine (V), leucine (L), alanine (A), or methionine (M) in a wide range of species among the clades. Given these substitutions that conserve the hydrophobic side chain, we hypothesize that all three residues are essential for protein structural folding. Another residue that appears to be conserved in all clades is Asp245, except for a substitution with a serine (Ser) in *Sporosarcina ureilytica* (second clade). The next residue, Glu 246, is entirely conserved in clades 3, 4, and 5. While in the other clades the presence of glutamic acid is still prevalent, it can be substituted in some species by alanine, threonine, valine, arginine, isoleucine, aspartic acid, glutamine, and serine.

Finally, Asn273 and Thr274 are fully conserved in clade 3, while Asn273 can be substituted for aspartic acid in a few species of clades 1, 2, 4, 5, 6, and 7, and for histidine in some species of clade 2. Thr274 is highly conserved in clades 3, 4, and 5, but can be substituted for a serine in clades 1, 2, and 7, for an isoleucine in clades 6 and 7, and for alanine, valine, or proline in clade 7.

### Docking Across Conformational Ensembles

To explore how small molecules may interact with the putative binding pocket identified in our structural analyses, we performed molecular docking using two previously reported Sa-CcpA inhibitors, Auranofin^38^ and TDP,^39^ as molecular probes to investigate potential ligand-binding modes within the proposed cavity. To ensure a comprehensive evaluation, we conducted a systematic docking study across multiple conformational states derived from cluster analysis of molecular dynamics trajectories (Figure S6). This ensemble-based approach enabled us to assess the robustness of the predicted pocket, determine how conformational dynamics influence ligand accommodation, and identify recurrent interaction conserved across distinct structural states.

Figure 7 shows the results of the docking analyses. As expected, both ligands displayed low score values for the crystallographic conformation, indicating that the cavity remained closed and was therefore unsuitable for ligand anchoring (Figure 7A). In contrast, the ligands were better accommodated in the conformations obtained from the analysis of the molecular dynamics trajectories, except for the most populated clusters of the monomer bound to DNA (monomer_DNA1) and the second most populated cluster of the dimer in the extended conformation (dimer_ext2). These results suggest that the region predicted as the ligand-binding site is stable and can accommodate inhibitors, including Auronafin, which is relatively bulky. Moreover, the docking scores showed no significant differences between monomeric and dimeric states (Table S3).

**Figure 7:**
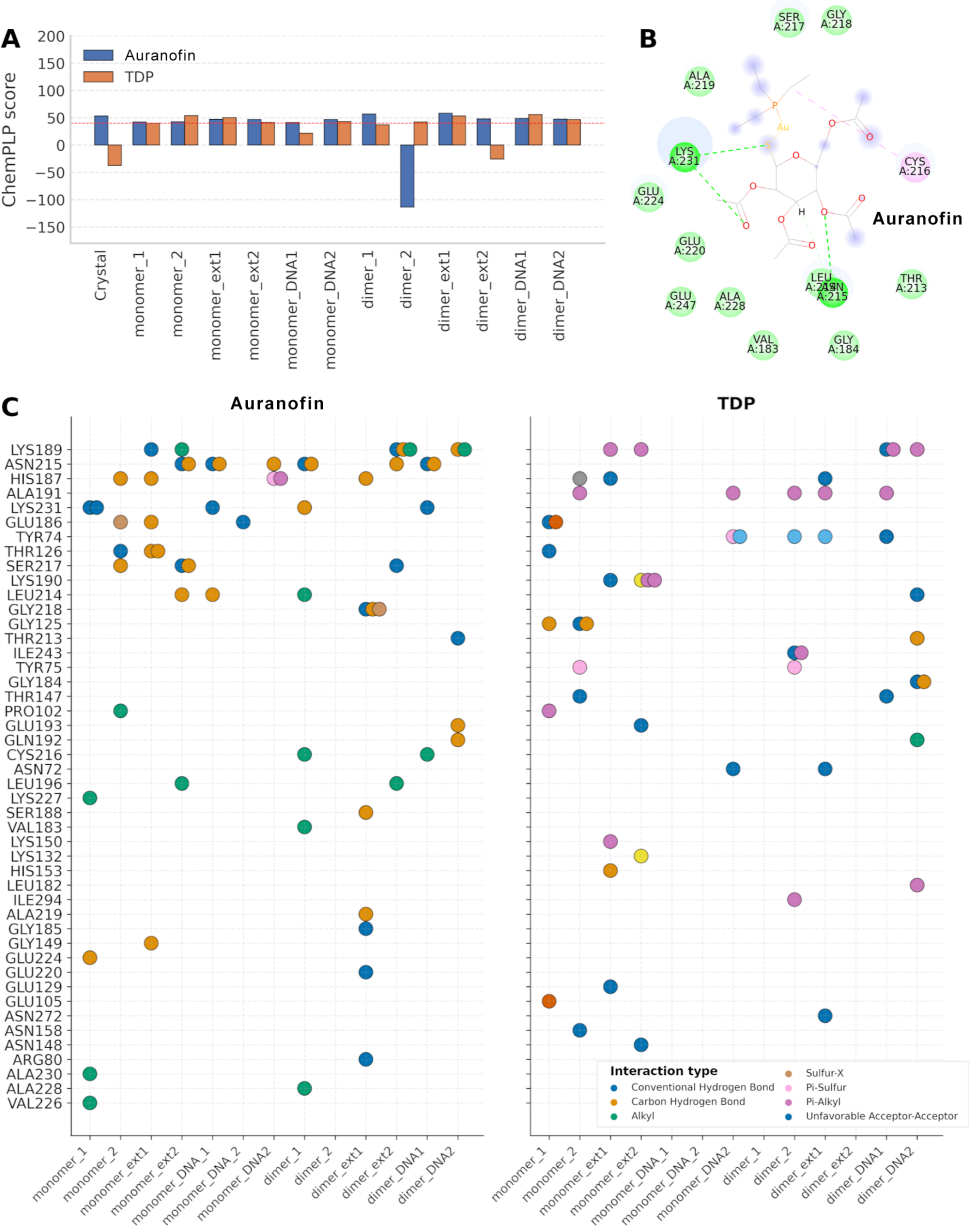
Probing TDP and Auranofin binding using molecular docking. Scoring function values for the best-ranked poses of Auranofin (blue) and TDP (orange) across the crystallographic structure (Crystal) and the two most representative clusters obtained for each condition: monomer (monomer_1 and monomer_2), monomer with an extended DBD (monomer_ext1 and monomer_ext2), monomer bound to DNA (monomer_DNA1 and monomer_DNA2), dimer (dimer_1 and dimer_2), dimer with an extended DBD (dimer_ext1 and dimer_ext2), and dimer bound to DNA (dimer_DNA1 and dimer_DNA2). The red dashed line indicates the threshold used to select poses for interaction analysis (A). Intermolecular interaction diagram of the best-ranked docking pose of Auronafin within the binding pocket of the dimeric DNA-bound form of CcpA, generated using Discovery Studio Visualizer (B). Interaction map summarizing the contacts formed between different CcpA conformations and the ligands Auronafin and TDP across the distinct conformational states of Sa-CcpA (C).

These findings suggest a hypothesis with potential therapeutic relevance. Because both inhibitors were able to accommodate within the predicted pocket across several monomeric conformations, with docking scores comparable to those of the dimeric structures, it is plausible that this region remains accessible prior to dimer formation. Since dimerization is required for DNA binding, perturbing this step could represent a strategy for modulating Sa-CcpA activity. In this context, ligands designed to bind residues within this pocket, including Cys216 and Cys242, might influence the conformational transitions necessary for functional dimerization. Notably, previous experimental studies have shown that these cysteines are chemically reactive and functionally critical (38, 40). These observations are consistent with our computational results and suggest that targeting this region may provide a viable avenue for regulating Sa-CcpA function. Therefore, we evaluated the interaction profiles of the best-ranked poses in the different conformational states (Figure 7B).

We observed a greater number of interactions, 14 and 13, in cluster 1 of the dimer and monomer trajectories, respectively. This indicates that the extended conformation provides the greatest pocket accessibility. Such accessibility strongly influences the ability of bulky ligands, including Auronafin, to interact with the protein, enabling them to form more contacts than in other conformational states. Among all the residues involved in ligand recognition, interactions with Lys189 were the most recurrent, appearing in nearly every condition for both ligands. Other frequently observed residues included Asn215, His187, Lys231, and Ala191. Regarding the predominant categories of interactions, Conventional Hydrogen Bonds were the most represented (38 interactions), followed by Carbon Hydrogen Bonds,^30^ Pi-Alkyl,^17^ and Alkyl.^14^

Representative docking poses of TDP and Auranofin within the predicted cavity of Sa-CcpA are shown in Figure 8, highlighting the residues involved in potential intermolecular interactions.

**Figure 8:**
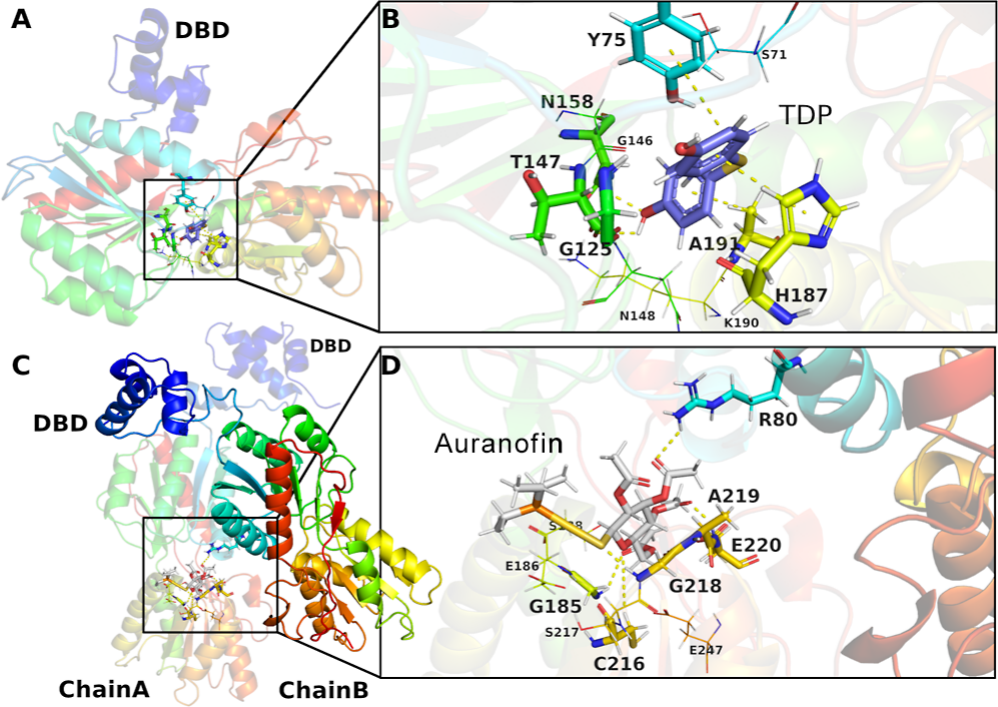
Representative docking poses of TDP and Auranofin in Sa-CcpA. Overall structure of the monomeric form of Sa-CcpA showing the DNA-binding domain and the location of the predicted cavity identified from the structural alignment (A). Detailed view of TDP within the cavity, highlighting residues involved in intermolecular interactions (B). Dimeric conformation of Sa-CcpA indicating the spatial arrangement of the two DBD and the location of the predicted cavity (C). Docking pose of Auranofin illustrating intermolecular interactions, including contacts with Cys216 and Arg80 from chain B (D). Intermolecular interactions are represented as yellow dashed lines and were visualized using PyMOL. Residues shown as sticks represent directional interactions, such as hydrogen bonds, whereas residues shown as lines represent van der Waals interactions.

Notably, Auronafin predominantly interacted with residues located closer to the dimerization region, including Lys189, Asn215, Ser217, Glu186, and Cys216, through hydrogen bonding, sulfur-related contacts, and hydrophobic interactions, whereas TDP formed hydrogen bonds with Asn72, His187, and others. Significant differences were also observed in the interaction profiles of the two inhibitors. Furthermore, a marked difference in ligand interaction patterns was observed when comparing the monomeric and dimeric forms. The dimer displayed unique interactions with residues Leu182, Leu214, Gly184, Gln192, Thr213, Asn272, Ser188, Gly124, Ile294, Ile243, and Gly149, highlighting conformational and structural features accessible only upon dimer formation.

## Conclusions

In this study, we developed a structural alignment-based strategy capable of identifying ligand-binding sites in the transcription factor CcpA from *S. aureus*, a protein crystallized in a closed conformation that prevents the detection of cavities using conventional binding site prediction methods. By integrating molecular dynamics simulations and docking analyses, we evaluated the conformational stability of a newly identified binding region and identified the key residues potentially involved in ligand recognition. Our results suggest the presence of a previously uncharacterized effector-binding region in Sa-CcpA, which may contribute to its regulation beyond canonical pathways such as copper-mediated cysteine oxidation.

In this context, docking analyses indicated that TDP can adopt plausible binding poses within this region. In contrast, docking of auranofin directed toward Cys216 suggested potential interactions with this residue, which were only observed in the dimeric conformation of CcpA, consistent with experimental evidence indicating the involvement of cysteine residues in auranofin activity. Notably, phylogenetic analysis revealed that several residues within this predicted binding site are conserved in a subset of pathogenic Bacillales, suggesting functional constraints relevant to species-specific regulatory roles.

Taken together, these findings illustrate the potential of this integrated approach to uncover inaccessible ligand-binding pockets in TFs with poorly defined regulatory sites. Although this strategy was implemented in the case study of Sa-CcpA, it may serve broader efforts in the structure-based design of inhibitors targeting transcription regulators in bacterial pathogens.

## Experimental Section

### Data Set and Structural Preparation

To obtain crystallographic structural models of bacterial transcription factors, we utilized the EntraF database.^63^ EntraF serves (https://entraf.iimas.unam.mx) as an encyclopedia of well-annotated DNA-binding transcription factors found in Bacteria and*Archaea*, encompassing proteins dedicated to regulate gene expression at the transcriptional level. Subsequent filtering was applied to the retrieved structures to isolate transcription factors cocrystallized with ligands. The study has carefully selected a total of 212 protein structures.

The initial step involved aligning the amino acid sequence of CcpA from *S. aureus*, strain Bmb93937 against the crystallographic structure deposited in the Protein Data Bank (PDB^64^) using the Blast^65^ tool (Basic Local Alignment Search Tool). After the BLASTp search, we selected the structure of Sa-CcpA (PDB ID: 7E5W,^40^ with a resolution of 2.5 Å). However, this crystal structure contains an unresolved region spanning residues 51–58. To address this, the missing region was modeled using the SWISS-MODEL server,^66^ employing the Sa-CcpA crystal structure itself as the template.

The geometric parameters of the modeled structure were validated with PROCHECK^67^ from the UCLA-DOE LAB server (saves.mbi.ucla.edu), which generated the Ramachandran plot (see Figure S1 in Supporting Information).

Given that several proteins within this family adopt an extended conformation of the DBD, we also generated a model of Sa-CcpA in this alternative state to assess potential conformational differences. The extended conformation was retrieved from the AlphaFold Protein Structure Database^68^ under the accession code P99175.

Additionally, a model of Sa-CcpA bound to the promoter region of *tst*, the gene encoding toxic shock syndrome toxin 1 (TSST-1),^69^ was constructed. The three-dimensional structure of the DNA sequence was generated using Discovery Studio Visualizer v24.1.0.23298,^70^ and subsequently used to assemble the protein–DNA complex.

All systems: (i) crystallographic, (ii) extended, and (iii) DNA-bound were prepared in both monomeric and dimeric forms.

### Sequence Alignment and Phylogenetic Tree

The reference genomes belonging to the order*Bacillales* (taxid: 1385) were retrieved from the NCBI, including the amino acid sequences of their annotated proteins. All files were organized in FASTA format, thereby forming the complete proteome of each organism included in the analysis. The Sa-CcpA sequence was compared against this local protein library using BLASTp.^65^ Alignments were exported in tabular format (outfmt 6) and processed in Python in order to obtain only hits with sequence identity above 40% and query coverage above 90%. The identifiers of the selected alignments were then used to retrieve the corresponding sequences from the local library. These proteins were concatenated into a single FASTA file, generating the sequence set for subsequent analysis. To remove redundancy and cluster highly similar proteins, CD-HIT^71^ was applied with a 90% identity threshold, producing a FASTA file containing the representative sequence of each cluster along with its composition. To assess the impact of redundancy reduction on the phylogenetic analysis, an additional data set was generated without CD-HIT clustering. In this case, BLASTp hits were filtered using a more stringent threshold of sequence identity above 60%, and query coverage above 90%, and all selected sequences were retained for downstream analyses.

Phylogenetic and molecular evolutionary analyses were performed using MEGA version 10.2.6 ^72^ based on a data set comprising 659 protein sequences. The phylogenetic tree was constructed using the Maximum Likelihood (ML) method with the LG substitution model. The rate variation among sites was modeled using a discrete Gamma distribution with invariant sites (G + I). The robustness of the inferred topology was assessed using the bootstrap method with 1000 replicas. The resulting tree was visualized and annotated using iTOL version 7.2.2.^73^

### Structural Alignment

Once we had obtained the structural models of the TFs altered in the biofilm and the TFs with cocrystallized ligands from the EntraF database, we loaded these structures into a molecular graphics system called PyMOL.^74^ Subsequently, we conducted structural alignments between all TFs in the biofilm and their counterparts in the EntraF database using the align, super, and cealign,^75^ which is an integrated analysis of PyMOL. Following structural alignment, we performed root-mean-square deviation (RMSD) calculations to assess the degree of alignment between the TFs. This workflow was fully automated using a Python script, which is available on the GitHub repository (https://github.com/farias-ab/TFStructAlign.git), along with the protein data sets and detailed instructions for reproducing all analyses.

### Pockets Detection

We employed a cavity detection strategy using the CavityPlus^45^ program in order to identify druggable pockets. CavityPlus is a web server package designed for the detection of cavities within protein structures, ranking them based on ligandability and druggability scores. We submitted the structure of Sa-CcpA obtained in PDB (7E5W), Ec-PurR (1BDH), and Bs-CcpA (1ZVV). The predicted cavities were further analyzed using PyMOL^74^ software (version 2.4.0a0).

### Molecular Docking

The ligands auranofin and TDP were built using the Maestro software,^76^ and their geometries were optimized with the semiempirical PM6 method implemented in Gaussian 09.^77^

Docking parameters were defined based on the analysis of poses obtained from the redocking of a structural homologue, PurR (PDB ID:1BDH, 2.7 Å resolution), which contains a cocrystallized ligand (hypoxanthine) in the same cavity corresponding to that of Sa-CcpA. The optimal parameters indicated the use of the ChemPLP scoring function with a 10 Å search radius centered on residue Arg190. Thirty genetic algorithm runs were performed for each molecule. The redocking parameters yielded an RMSD value of 0.284 Å between the cocrystallized ligand and the best pose, validating the docking protocol.

However, experimental evidence indicates that auranofin interacts with residues Cys216 and Cys242.^38^ In this case, an additional site-directed docking calculation was performed specifically for auranofin, using the oxygen atom of Cys216 as the center of the docking grid while maintaining the same docking parameters described above.

Subsequently, docking of the ligands was performed on the crystallographic structure of Sa-CcpA (PDB ID: 7E5W), using the GOLD v.2024.3.0 software.^78^ The resulting poses were analyzed using the default parameters of Discovery Studio Visualizer v24.1.0.23298 ^70^ to identify and classify the types of intermolecular interactions. Docking studies were also carried out on the two most representative clusters from the concatenated trajectories obtained from molecular dynamics simulations of the following systems: (i) monomer, (ii) dimer, (iii) extended monomer, (iv) extended dimer, (v) monomer bound to DNA, and (vi) dimer bound to DNA.

### Molecular Dynamics Simulations and Analysis

In this study, we conducted multiple molecular dynamics simulations using the Gromacs^79^ program (v. 2020.2). The force field used was AMBER99SB,^80^ in combination with the TIP3P^81^ water model. Simulations were performed under periodic boundary conditions, employing cubic boxes with a 1 nm distance between the solute and the box. To ensure system neutrality, we introduced NA^+^ and Cl^–^ ions.

For integration, we employed a leapfrog scheme with a time step of 2 fs. All bonds involving hydrogen atoms were constrained using the LINCS^82^ procedure. A cutoff distance of 1.2 nm was applied to truncate the Lennard-Jones interactions and define the real-space region for electrostatic interactions. We utilized the Smooth Particle Mesh Ewald (SPME)^83^ method with a grid spacing of 0.12 nm. Pair lists were generated and updated following the Verlet^84^ scheme.

The simulations began with energy minimization, involving 50,000 steps of the steepest descent algorithm. Subsequently, an equilibration was performed. During equilibration, positional restraints were applied to the protein atoms, and the system was gradually heated from 50 to 300 K over 1.2 ns in 50 K increments. The simulations were executed under NPT conditions, with a reference pressure of 1 bar and a reference temperature of 310 K. Temperature control was achieved using a velocity-rescaling algorithm,^85^ with separate coupling of the protein and solvent degrees of freedom to temperature baths, employing a coupling constant of 0.1 ps. Pressure was maintained near the reference value using the Parrinello–Rahman barostat,^86^ with an isothermal compressibility of 4.5 × 10^–5^ bar^–1^. Molecular dynamics production runs were performed in triplicate, each extending for 200 ns.

Cluster analysis is commonly employed to group similar conformations into subgroups called clusters. This analysis can identify the most stable conformations sampled throughout the simulation. In this work, we concatenated the last 100 ns of each trajectory from the three replicas and performed clustering using the GROMOS method with cutoffs of 0.125, 0.150, 0.180, and 0.210. The cutoff values were selected based on the distribution of the number of clusters generated for each threshold. The optimal cutoff will be the one that presents the best distribution of conformations per cluster, the first four clusters should contain 90% of the trajectory, and there should not be many clusters with one component (Figures S6).

We also performed an analysis of the Solvent Accessible Surface Area (SASA) of the predicted cavity throughout the MD trajectory. The cavity was defined as the set of residues located within a 10 Å radius from the ligand positions obtained after structural alignment of transcription factor complexes from the PDB entries 1BDH, 1EFA, and 2PE5 (Figure 5). These residues were then used to monitor the SASA variation along the trajectory, allowing us to quantify changes in pocket exposure during the simulation.

The conformational dynamics of the protein along the trajectory was explored through principal component analysis (PCA) to reveal the most dominant and collective motions. For this purpose, we concatenated the last 100 ns of trajectory obtained from the three replicas for each Sa-CcpA system: (i) the crystallographic structure, (ii) the extended DBD conformation, and (iii) the protein bound to DNA. The PCA analysis was performed for both the monomer and dimer Sa-CcpA system. The dominant modes were obtained from the diagonalization of the covariance matrix calculated for the Cα atoms over 300 ns of the trajectory. This procedure yielded eigenvectors and eigenvalues, which represent the directions of motion and the corresponding amplitudes, respectively. The GROMACS module *covar* was employed to compute the covariance matrix, while the anaeig module was used to analyze the resulting eigenvalues and eigenvectors.

Initially, all trajectories were concatenated and subjected to principal component analysis (PCA), as previously described. The first two principal components (PC1 and PC2), which account for the majority of the system’s conformational variation, were employed as collective coordinates to construct the free energy landscape (FEL), thus illustrating energetically favorable conformations and potential state transitions. The free energy was estimated from the distribution of states using the Boltzmann relation. For each replica, a FEL map was generated using the same PC1–PC2 mesh and energy scale, allowing direct comparison between replicates and systems. The global energy minimum in each replica was automatically identified from the lowest free-energy point and highlighted on the corresponding map with distinct symbols.

## Supplementary Material



## Data Availability

Detailed descriptions of the structural alignment workflow, input data sets, source code of all Python scripts, instructions for execution and the complete collection of annotated transcription factors used in this study are available in the GitHub repository: https://github.com/farias-ab/TFStructAlign.git. Additionally, the collection of regulatory proteins and the associated information used to guide the identification of ligand-bound transcription factors are freely accessible through the EntraF server (https://entraf.iimas.unam.mx).

## Notes

Detailed descriptions of Figures S1 to S10 (PDF)

## References

[1] Heaton C. J., Gerbig G. R., Sensius L. D., Patel V., Smith T. C. (2020). Staphylococcus
aureus Epidemiology in Wildlife: A Systematic Review. Antibiotics.

[2] Daum R. S. (2007). Skin and
Soft-Tissue Infections Caused by Methicillin-Resistant *Staphylococcus
aureus*. N. Engl. J. Med..

[3] Asgeirsson H., Thalme A., Weiland O. (2018). *Staphylococcus
aureus* bacteraemia and endocarditis – epidemiology
and outcome:
a review. Infect. Dis..

[4] Nasser A., Azimi T., Ostadmohammadi S., Ostadmohammadi S. (2020). A comprehensive
review of bacterial osteomyelitis with emphasis on Staphylococcus
aureus. Microb. Pathog..

[5] Horino T., Hori S. (2020). Metastatic infection during Staphylococcus aureus bacteremia. J. Infect. Chemother..

[6] Kim C.-J. (2019). Impact of antimicrobial
treatment duration on outcome of Staphylococcus
aureus bacteraemia: a cohort study. Clin. Microbiol.
Infect..

[7] Rubinstein E., Kollef M., Nathwani D. (2008). Pneumonia
Caused by Methicillin-Resistant *Staphylococcus aureus*. Clin. Infect.
Dis..

[8] De
La Calle C., Morata L., Cobos-Trigueros N., Martinez J. A., Cardozo C., Mensa J., Soriano A. (2016). Staphylococcus
aureus bacteremic pneumonia. Eur. J. Clin. Microbiol.
Infect. Dis..

[9] Suleiman A. S., Bhattacharya P., Islam M. A. (2025). Global prevalence and dynamics of
mecA and mecC genes in MRSA: Meta-meta-analysis, meta-regression,
and temporal investigation. Journal of Infection
and Public Health.

[10] Mlynarczyk-Bonikowska B., Kowalewski C., Krolak-Ulinska A., Marusza W. (2022). Molecular Mechanisms
of Drug Resistance in Staphylococcus aureus. Int. J. Mol. Sci..

[11] Xia Y., Hu Z., Jin Q., Chen Q., Zhao C., Qiang R., Xie Z., Li L., Zhang H. (2025). Structural characteristics, functions,
and counteracting strategies of biofilms in Staphylococcus aureus. Comput. Struct. Biotechnol. J..

[12] Wang D., Wang L., Liu Q., Zhao Y. (2025). Virulence
factors in
biofilm formation and therapeutic strategies for Staphylococcus aureus:
A review. Anim. Zoonoses.

[13] Brüssow H. (2024). The antibiotic
resistance crisis and the development of new antibiotics. Microb. Biotechnol..

[14] World Health Organization . 2023 Antibacterial agents in clinical and preclinical development: an overview and analysis; World Health Organization. 2024.

[15] Mitsakakis K., Kaman W. E., Elshout G., Specht M., Hays J. P. (2018). Challenges
in Identifying Antibiotic Resistance Targets for Point-of-Care Diagnostics
in General Practice. Future Microbiol..

[16] Peraman R., Sure S. K., Dusthackeer V. N. A., Chilamakuru N. B., Yiragamreddy P. R., Pokuri C., Kutagulla V. K., Chinni S. (2021). Insights on recent
approaches in drug discovery strategies
and untapped drug targets against drug resistance. Future Journal of Pharmaceutical Sciences.

[17] da
Cunha B. R., Fonseca L. P., Calado C. R. C. (2019). Antibiotic Discovery:
Where Have We Come from, Where Do We Go?. Antibiotics.

[18] Serral F., Castello F. A., Sosa E. J., Pardo A. M., Palumbo M. C., Modenutti C., Palomino M. M., Lazarowski A., Auzmendi J., Ramos P. I. P., Nicolás M. F., Turjanski A. G., Martí M. A., Fernández Do Porto D. (2021). From Genome
to Drugs: New Approaches in Antimicrobial Discovery. Front. Pharmacol..

[19] Da Silva, M. M. P. ; Guedes, I. A. ; Custódio, F. L. ; Da Silva, E. K. ; Dardenne, L. E. In Structure-Based Drug Design; Marti, M. A. ; Turjanski, A. G. ; Fernández Do Porto, D. , Eds.; Computer-Aided Drug Discovery and Design; Springer International Publishing: Cham, 2024; Vol. 2; pp 177–221.

[20] Krishnan A. (2025). A generative deep learning approach to de novo
antibiotic design. Cell.

[21] Stokes J. M. (2020). A Deep Learning Approach to Antibiotic Discovery. Cell.

[22] Wang Z., Koirala B., Hernandez Y., Zimmerman M., Brady S. F. (2022). Bioinformatic prospecting and synthesis
of a bifunctional
lipopeptide antibiotic that evades resistance. Science.

[23] Das P., Sercu T., Wadhawan K., Padhi I., Gehrmann S., Cipcigan F., Chenthamarakshan V., Strobelt H., Dos Santos C., Chen P.-Y., Yang Y. Y., Tan J. P. K., Hedrick J., Crain J., Mojsilovic A. (2021). Accelerated
antimicrobial discovery
via deep generative models and molecular dynamics simulations. Nat. Biomed. Eng..

[24] Cortés-Avalos D., Borges Farias A., Romero-González L. E., Lara-Ochoa C., Villa-Tanaca L., García-del Portillo F., López-Guerrero V., Bustamante V. H., Pérez-Rueda E., Ibarra J. A. (2024). Interactions between
the AraC/XylS-like transcriptional activator InvF of Salmonella Typhimurium,
the RNA polymerase alpha subunit and the chaperone SicA. Sci. Rep..

[25] Farias A. B., Cortés-Avalos D., Ibarra J. A., Perez-Rueda E. (2024). The interaction
of InvF-RNAP is mediated by the chaperone SicA in *Salmonella* sp: an *in silico* prediction. PeerJ.

[26] Farias A. B., Candiotto G., Siragusa L., Goracci L., Cruciani G., Oliveira E. R. A., Horta B. A. C. (2021). Targeting Nsp9
as an anti-SARS-CoV-2
strategy. New J. Chem..

[27] Balleza E., López-Bojorquez L. N., Martínez-Antonio A., Resendis-Antonio O., Lozada-Chávez I., Balderas-Martínez Y. I., Encarnación S., Collado-Vides J. (2009). Regulation by transcription factors
in bacteria: beyond description. FEMS Microbiol.
Rev..

[28] Agris P. F. (2025). Targeting
Gene Transcription Prevents Antibiotic Resistance. Antibiotics.

[29] Zhuang J.-j., Liu Q., Wu D.-l., Tie L. (2022). Current strategies and progress for
targeting the “undruggable” transcription factors. Acta Pharmacol. Sin..

[30] Yang Y., Zhang L., Huang H., Yang C., Yang S., Gu Y., Jiang W. (2017). A Flexible Binding Site Architecture Provides New Insights
into CcpA Global Regulation in Gram-Positive Bacteria. mBio.

[31] Seidl K., Stucki M., Ruegg M., Goerke C., Wolz C., Harris L., Berger-Bächi B., Bischoff M. (2006). *Staphylococcus
aureus* CcpA Affects Virulence Determinant Production and
Antibiotic Resistance. Antimicrob. Agents Chemother..

[32] Bulock L. L., Ahn J., Shinde D., Pandey S., Sarmiento C., Thomas V. C., Guda C., Bayles K. W., Sadykov M. R. (2022). Interplay
of CodY and CcpA in Regulating Central Metabolism and Biofilm Formation
in Staphylococcus aureus. J. Bacteriol..

[33] Zheng M., Zhu K., Peng H., Shang W., Zhao Y., Lu S., Rao X., Li M., Zhou R., Li G. (2022). CcpA Regulates Staphylococcus
aureus Biofilm Formation through Direct Repression of Staphylokinase
Expression. Antibiotics.

[34] Urso A. (2024). Staphylococcus aureus adapts to exploit collagen-derived proline
during chronic infection. Nat. Microbiol..

[35] Seidl K., Goerke C., Wolz C., Mack D., Berger-Bächi B., Bischoff M. (2008). *Staphylococcus
aureus* CcpA Affects
Biofilm Formation. Infect. Immun..

[36] Costa M. D. O. C. E., Nascimento A. P. B. D., Martins Y. C., Santos M. T. D., Figueiredo A. M. D. S., Perez-Rueda E., Nicolás M. F. (2023). The gene regulatory network of Staphylococcus aureus
ST239-SCCmecIII strain Bmb9393 and assessment of genes associated
with the biofilm in diverse backgrounds. Front.
Microbiol..

[37] Schumacher M. A., Sprehe M., Bartholomae M., Hillen W., Brennan R. G. (2011). Structures
of carbon catabolite protein A–(HPr-Ser46-P) bound to diverse
catabolite response element sites reveal the basis for high-affinity
binding to degenerate DNA operators. Nucleic
Acids Res..

[38] Lin W., Chen J., Huang Z., Li H., Chen Y., Duan X., Xiong Y., Han B., Jiang G., Wang J., Liao X. (2025). Targeting catabolite
control protein
A in *Staphylococcus aureus* with auranofin. Inorg. Chem. Front..

[39] Huang Q., Zhang Z., Li H., Guo Y., Liao X., Li H., Zhou H., Xia W. (2020). Identification
of a Novel Inhibitor
of Catabolite Control Protein A from *Staphylococcus aureus*. ACS Infect. Dis..

[40] Liao X., Li H., Guo Y., Yang F., Chen Y., He X., Li H., Xia W., Mao Z.-W., Sun H. (2022). Regulation of DNA-binding
activity of the Staphylococcus aureus catabolite control protein A
by copper (II)-mediated oxidation. J. Biol.
Chem..

[41] Schumacher M. A., Seidel G., Hillen W., Brennan R. G. (2006). Phosphoprotein Crh-Ser46-P
Displays Altered Binding to CcpA to Effect Carbon Catabolite Regulation. J. Biol. Chem..

[42] Glasfeld A., Schumacher M. A., Choi K.-Y., Zalkin H., Brennan R. G. (1996). A Positively
Charged Residue Bound in the Minor Groove Does Not Alter the Bending
of a DNA Duplex. J. Am. Chem. Soc..

[43] Wu S., Wang F., Zhou W., Zhang X., Zhan L., Zhang W., Wang W., Zhang W., Huang S., Fernie A. R., Liu Z., Yan S. (2025). Conformational plasticity
of disordered regions enables sequence-diverse DNA recognition by
transcription factor AflR. Nat. Commun..

[44] Jain D., Narayanan N., Nair D. T. (2016). Plasticity in Repressor-DNA
Interactions
Neutralizes Loss of Symmetry in Bipartite Operators. J. Biol. Chem..

[45] Xu Y., Wang S., Hu Q., Gao S., Ma X., Zhang W., Shen Y., Chen F., Lai L., Pei J. (2018). CavityPlus: a web server
for protein cavity detection with pharmacophore
modelling, allosteric site identification and covalent ligand binding
ability prediction. Nucleic Acids Res..

[46] Hark
Gan H., Perlow R. A., Roy S., Ko J., Wu M., Huang J., Yan S., Nicoletta A., Vafai J., Sun D., Wang L., Noah J. E., Pasquali S., Schlick T. (2002). Analysis of Protein Sequence/Structure
Similarity Relationships. Biophys. J..

[47] Schumacher M. A., Allen G. S., Diel M., Seidel G., Hillen W., Brennan R. G. (2004). Structural Basis
for Allosteric Control of the Transcription
Regulator CcpA by the Phosphoprotein HPr-Ser46-P. Cell.

[48] Szarvas J., Nag S., Otani S., Birkedahl L. E. K., Møller F. D., Asante-Sefa S., Daley D., Gustafson N. W., Møller M., Onipede A., Tafaj S., Aarestrup F. M. (2024). Complete
genome sequences of Cytobacillus sp., Domibacillus sp., Enterobacter
sp., *Neisseria* sp., *Pseudomonas* sp.,
and *Streptococcus* sp. strains from human clinical
infections collected at diagnostic units in 2020. Microbiol. Resour. Announce..

[49] Kim S. G., Summage-West C. V., Reyna M., Kim S.-J., Foley S. L. (2022). Complete
Genome Sequence of Metabacillus litoralis Strain NCTR108, Isolated
from Commercial Tattoo Ink. Microbiol. Resour.
Announce..

[50] Leiba J., Hartmann T., Cluzel M.-E., Cohen-Gonsaud M., Delolme F., Bischoff M., Molle V. (2012). A Novel Mode
of Regulation
of the Staphylococcus aureus Catabolite Control Protein A (CcpA) Mediated
by Stk1 Protein Phosphorylation. J. Biol. Chem..

[51] Beltramini A. M., Mukhopadhyay C. D., Pancholi V. (2009). Modulation of Cell
Wall Structure
and Antimicrobial Susceptibility by a *Staphylococcus aureus* Eukaryote-Like Serine/Threonine Kinase and Phosphatase. Infect. Immun..

[52] Akoua-Koffi C., Kacou N’Douba A., Djaman J. A., Herrmann M., Schaumburg F., Niemann S. (2022). Staphylococcus schweitzeri—An Emerging One Health
Pathogen?. Microorganisms.

[53] Chen S.-Y., Lee H., Wang X.-M., Lee T.-F., Liao C.-H., Teng L.-J., Hsueh P.-R. (2018). High mortality impact of Staphylococcus argenteus on
patients with community-onset staphylococcal bacteraemia. Int. J. Antimicrob. Agents.

[54] Rocha
Balzan L. D. L., Rossato A. M., Riche C. V. W., Cantarelli V. V., D’Azevedo P. A., Valerio De Lima A., Rodrigues B., Franca E Silva I. L. A., Dias C. A. G., Sampaio J. L. M. (2023). *Staphylococcus
argenteus* Infections, Brazil. Microbiol.
Spectrum.

[55] Al
Masalma M., Raoult D., Roux V. (2010). Staphylococcus massiliensis
sp. nov., isolated from a human brain abscess. Int. J. Syst. Evol. Microbiol..

[56] Maslanova I., Wertheimer Z., Sedláček I., Švec P., Indráková A., Kovařovic V., Schumann P., Spröer C., Králová S., Šedo O. (2018). Description and Comparative Genomics of *Macrococcus* caseolyticus subsp. hominis subsp. nov., *Macrococcus* goetzii sp. nov., *Macrococcus* epidermidis sp. nov., and *Macrococcus* bohemicus
sp. nov. Front. Microbiol..

[57] Belhout C., Wang F., Rossano A., Collaud A., Fernandez J. E., Marchionatti E., Keller J. E., Overesch G., Kaessmeyer S., Schwendener S., Perreten V. (2025). Macrococcus animalis sp. nov. and *Macrococcus* equi sp. nov., isolated from different animals’
origins. Int. J. Syst. Evol. Microbiol..

[58] Carroll L. M., Pierneef R., Mafuna T., Magwedere K., Matle I. (2023). Genus-wide genomic characterization of *Macrococcus*: insights into evolution, population structure, and functional potential. Front. Microbiol..

[59] Freu G., Gioia G., Gross B., Biscarini F., Virkler P., Watters R., Addis M. F., Franklin-Guild R. J., Runyan J., Masroure A. J., Bronzo V., Dos Santos M. V., Moroni P. (2024). Frequency of non-aureus staphylococci
and mammaliicocci
species isolated from quarter clinical mastitis: A 6-year retrospective
study. J. Dairy Sci..

[60] De
Carvalho T. P., Moreira L. G. A., Vieira A. D., Da Silva L. A., Santana C. H., Dos Santos D. O., Oliveira A. R., Tinoco H. P., Coelho C. M., Xavier R. G. C., Silva R. O. S., Da
Paixao T. A., Santos R. L. (2022). Mammaliicoccus (Staphylococcus) *sciuri*-induced suppurative meningoencephalitis and bacteremia
in an infant western lowland gorilla (*Gorilla gorilla gorilla*). J. Med. Primatol..

[61] Ye F., Lin Y., Li Y., Tu Y., Yang X., Han Y. (2025). Genomic characterization
of a multidrug-resistant Mammaliicoccus sciuri strain isolated from
urine of a patient from China. J. Antimicrob.
Chemother..

[62] Burke O. ´., Zeden M. S., O’Gara J. P. (2024). The pathogenicity
and virulence of
the opportunistic pathogen *Staphylococcus epidermidis*. Virulence.

[63] Tenorio-Salgado, S. ; Maya, C. R. ; Galan-Vasquez, E. ; Farias, A. B. ; Álvarez López, D. ; Villalpando-Aguilar, J. L. ; Martin, A. J. ; Ledesma-Dominguez, L. ; Perez-Rueda, E. ENcyclopedia of TRAnscription Factors in Bacteria and Archaea genomes (ENTRAF), Version 2.0, 2025. 10.1093/database/baaf071 PMC12569306 41158063

[64] Berman H. M. (2000). The Protein
Data Bank. Nucleic Acids Res..

[65] Altschul S. F., Gish W., Miller W., Myers E. W., Lipman D. J. (1990). Basic local
alignment search tool. J. Mol. Biol..

[66] Waterhouse A., Bertoni M., Bienert S., Studer G., Tauriello G., Gumienny R., Heer F. T., de Beer T. A. P., Rempfer C., Bordoli L., Lepore R., Schwede T. (2018). SWISS-MODEL: homology
modelling of protein structures and complexes. Nucleic Acids Res..

[67] Laskowski R. A., MacArthur M. W., Moss D. S., Thornton J. M. (1993). PROCHECK: a program
to check the stereochemical quality of protein structures. J. Appl. Crystallogr..

[68] Varadi M. (2022). AlphaFold Protein Structure
Database: massively expanding the structural
coverage of protein-sequence space with high-accuracy models. Nucleic Acids Res..

[69] Seidl K., Bischoff M., Berger-Bächi B. (2008). CcpA Mediates the Catabolite Repression
of tst in Staphylococcus aureus. Infect. Immun..

[70] BIOVIA Discovery Studio . Discovery Studio Modeling Environment. Version 21.1.0.20298; Dassault Systèmes: San Diego, 2016.

[71] Fu L., Niu B., Zhu Z., Wu S., Li W. (2012). CD-HIT: accelerated
for clustering the next-generation sequencing data. Bioinformatics.

[72] Kumar S., Stecher G., Li M., Knyaz C., Tamura K. (2018). MEGA X: Molecular
Evolutionary Genetics Analysis across Computing Platforms. Mol. Biol. Evol..

[73] Letunic I., Bork P. (2024). Interactive Tree of Life (iTOL) v6: recent updates to the phylogenetic
tree display and annotation tool. Nucleic Acids
Res..

[74] Schrodinger, L. The PyMOL molecular graphics system. Version 2.4.0a0 1, 0, 2010.

[75] Shindyalov I. N., Bourne P. E. (1998). Protein structure
alignment by incremental combinatorial
extension (CE) of the optimal path. Protein
Engineering Design and Selection.

[76] Schrodinger LLC. Schrodinger Release 2025–4: Maestro, 2025.

[77] Frisch, M. J. ; Gaussian 09, Revision E.01; Gaussian Inc.: Wallingford CT, 2009.

[78] Jones G., Willett P., Glen R. C., Leach A. R., Taylor R. (1997). Development
and validation of a genetic algorithm for flexible docking. J. Mol. Biol..

[79] Van
Der Spoel D., Lindahl E., Hess B., Groenhof G., Mark A. E., Berendsen H. J. C. (2005). GROMACS: Fast, flexible, and free. J. Comput. Chem..

[80] Hornak V., Abel R., Okur A., Strockbine B., Roitberg A., Simmerling C. (2006). Comparison
of multiple Amber force
fields and development of improved protein backbone parameters. Proteins: Struct., Funct., Bioinf..

[81] Jorgensen W. L., Chandrasekhar J., Madura J. D., Impey R. W., Klein M. L. (1983). Comparison
of simple potential functions for simulating liquid water. J. Chem. Phys..

[82] Hess B., Bekker H., Berendsen H. J. C., Fraaije J. G. E. M. (1997). LINCS: A linear
constraint solver for molecular simulations. J. Comput. Chem..

[83] Essmann U., Perera L., Berkowitz M. L., Darden T., Lee H., Pedersen L. G. (1995). A smooth particle
mesh Ewald method. J. Chem. Phys..

[84] Páll S., Hess B. (2013). A flexible algorithm for calculating pair interactions on SIMD architectures. Comput. Phys. Commun..

[85] Bussi G., Donadio D., Parrinello M. (2007). Canonical sampling through velocity
rescaling. J. Chem. Phys..

[86] Parrinello M., Rahman A. (1980). Crystal Structure
and Pair Potentials: A Molecular-Dynamics
Study. Phys. Rev. Lett..

