# Supplementary material for: Structure-Based Strategy Reveals a Novel Ligand Binding Site in Staphylococcus aureus Catabolite Control Protein A: Implications for Transcriptional Regulation and Drug Design
Source: ACS Omega. 2026 May 14;11(20):29817–30. doi: 10.1021/acsomega.6c00305 (PMC13216934; doi:10.1021/acsomega.6c00305)
Supplement: Supplementary file 1 [file ao6c00305_si_001.pdf]

# Supporting Information: Structure-based strategy reveals a novel ligand binding site in Staphylococcus aureus catabolite control protein A: implications for transcriptional regulation and drug design

André Borges Farias,[*,†] Maria Carolina Sisco,[†] Maiana de Oliveira Cerqueira e Costa,[†] Ernesto Perez-Rueda,[‡] and Marisa Fabiana Nicolás[*,†]

†*Laboratório de Bioinformática, Laboratório Nacional de Computação Científica (LNCC), Petrópolis, Rio de Janeiro, Brazil*

‡*Instituto de Investigaciones en Matemáticas Aplicadas y en Sistemas, Universidad Nacional Autónoma de México (UNAM), Unidad Académica del Estado de Yucatán, Mérida, Yucatán, México.*

E-mail: fariasab@lncc.br; marisa@lncc.br

# Figures

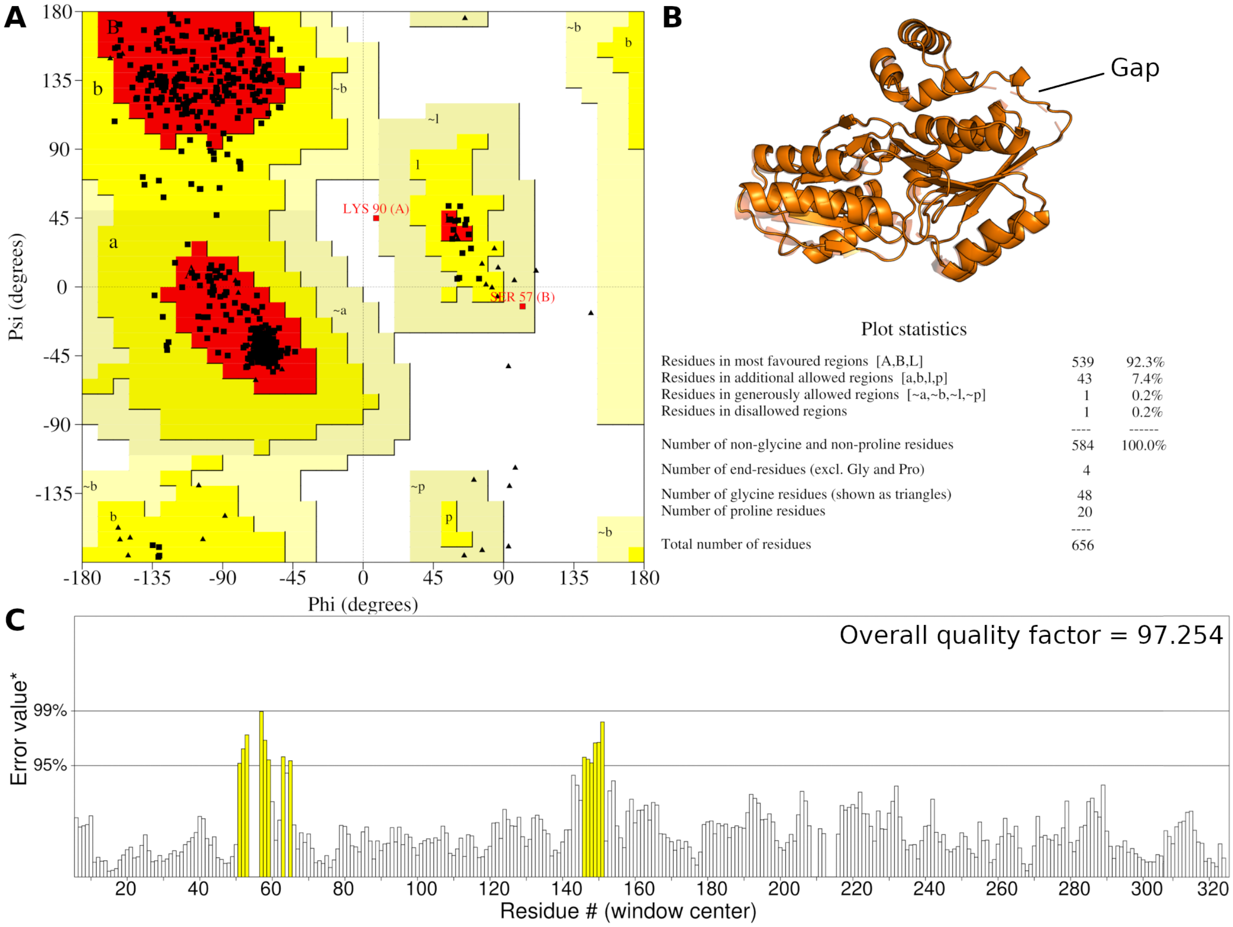


Figure S1: **Model validation of Sa-CcpA**. Ramachandran plot of Sa-CcpA (**A**), showing the original structure with the missing region highlighted in transparent and the modeled region in orange. Percentage of residues in the favored, allowed, and disallowed regions of the Ramachandran plot (**B**). ERRAT quality assessment plot displaying the residue error function (**C**).

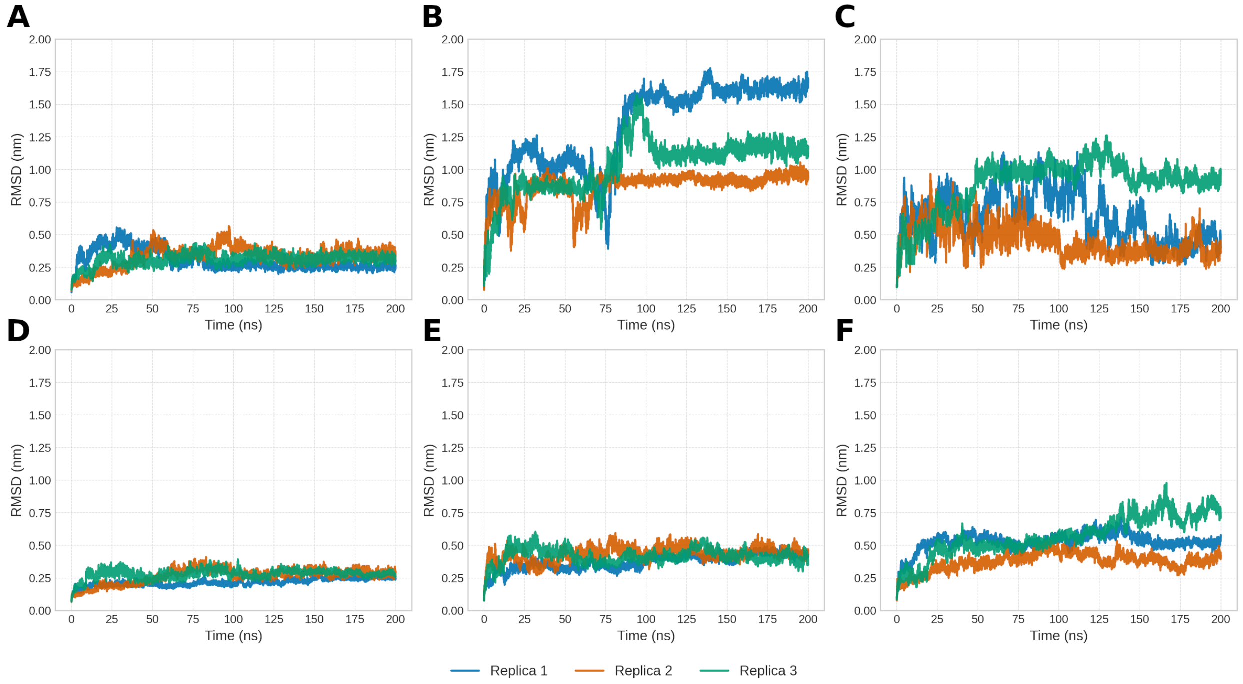


Figure S2: **Structural stability of Sa-CcpA over 200 ns of molecular dynamics simulation.** RMSD profiles of the (**A**) monomer, (**B**) monomer with DBD extended, (**C**) monomer with DNA, (**D**) dimer, (**E**) dimer with DBD extended, and (**F**) dimer with DNA.

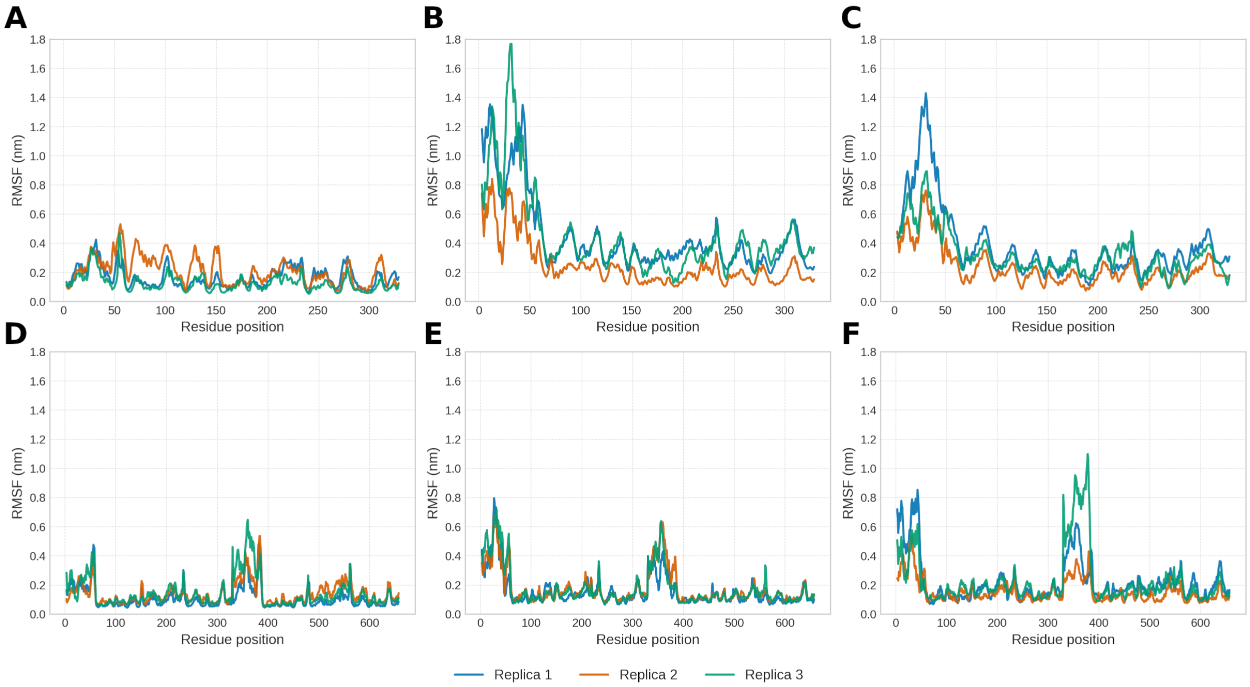


Figure S3: **Structural flexibility of Sa-CcpA residues throughout molecular dynamics simulations.** RMSF profiles of the (**A**) monomer, (**B**) monomer with DBD extended, (**C**) monomer with DNA, (**D**) dimer , (**E**) dimer with DBD extended, and (**F**) dimer with DNA, highlighting residue-specific flexibility, with peaks corresponding to regions exhibiting higher mobility during the simulations.

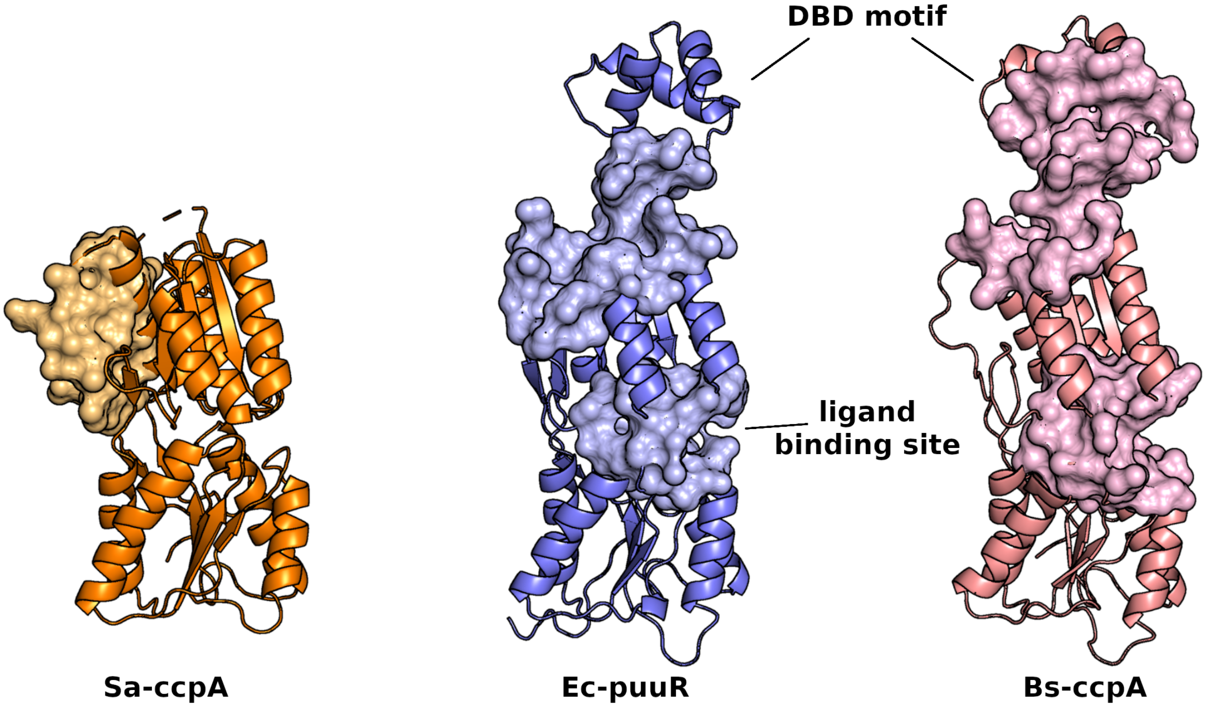


Figure S4: **Predicted druggable cavities in the proteins Sa-CcpA (left), Ec-PurR (middle) and Bs-CcpA (right), identified with CavityPlus.** Protein surfaces are displayed for top ranked cavities, indicating regions with highest binding potential for small molecules.

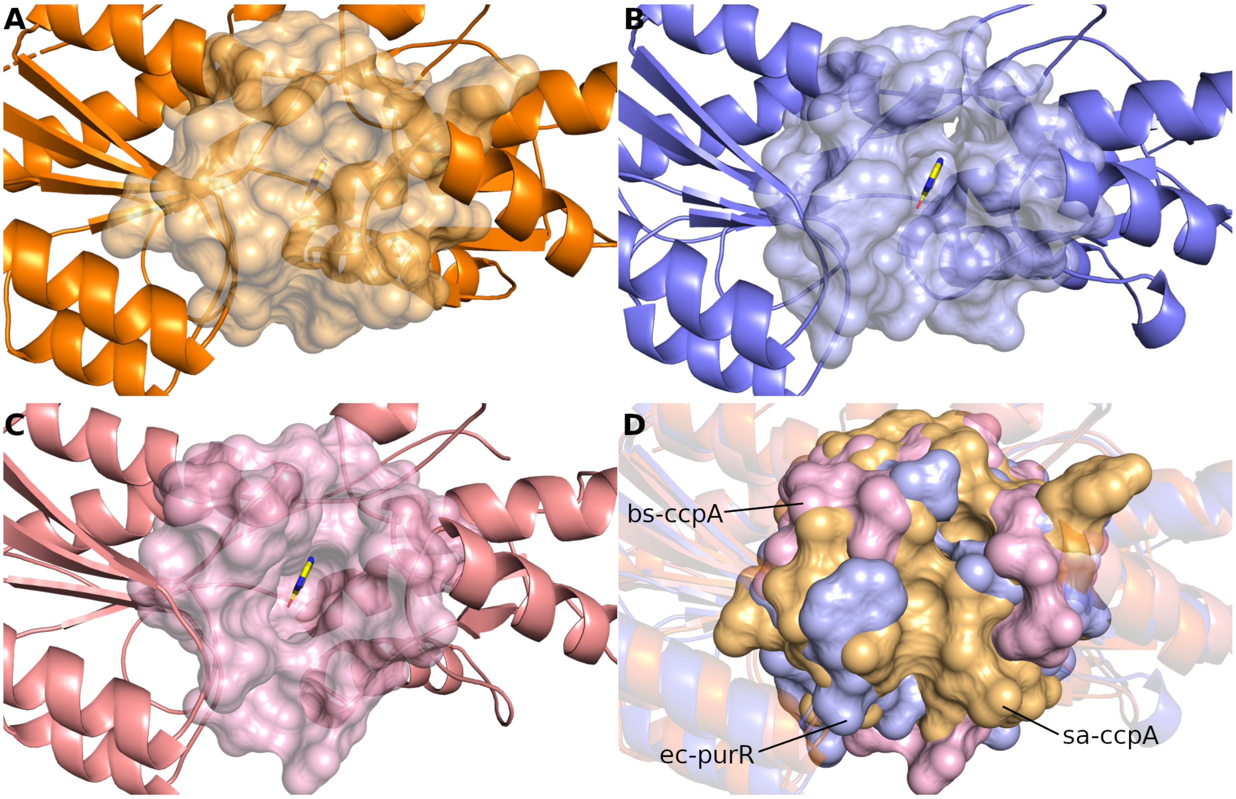


Figure S5: **Selection of all residues within a 10 Å radius of the ligand identified in the predicted cavity.** Molecular surfaces representations of Sa-CcpA (**A**), Ec-PurR (**B**), Bs-CcpA (**C**), and the overlap of the three cavities (**D**). The cavity searching approach for Sa-CcpA is not feasible due to its closed conformation.

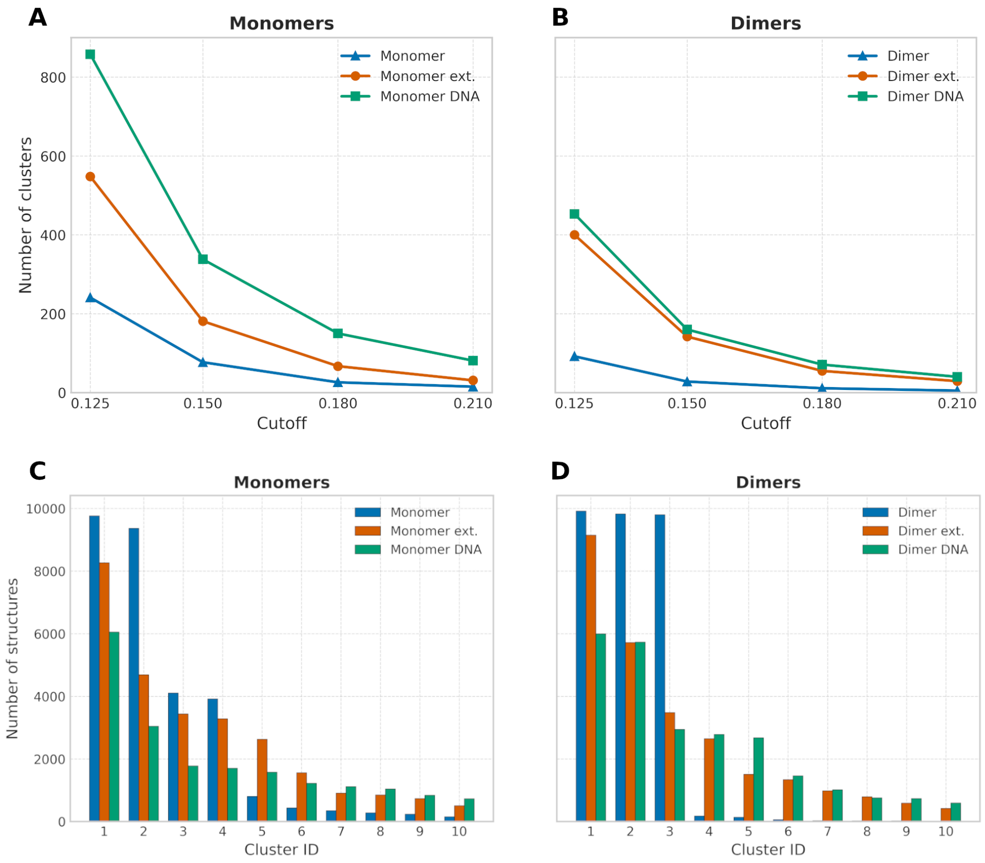


Figure S6: **Cluster analysis of the concatenated trajectories obtained for the monomer and dimer.** Number of clusters identified from the molecular dynamics trajectories as a function of the applied cutoff (**A**, monomer; **B**, dimer). Distribution of structures per cluster using a cutoff of 0.180 (**C**, monomer; **D**, dimer).

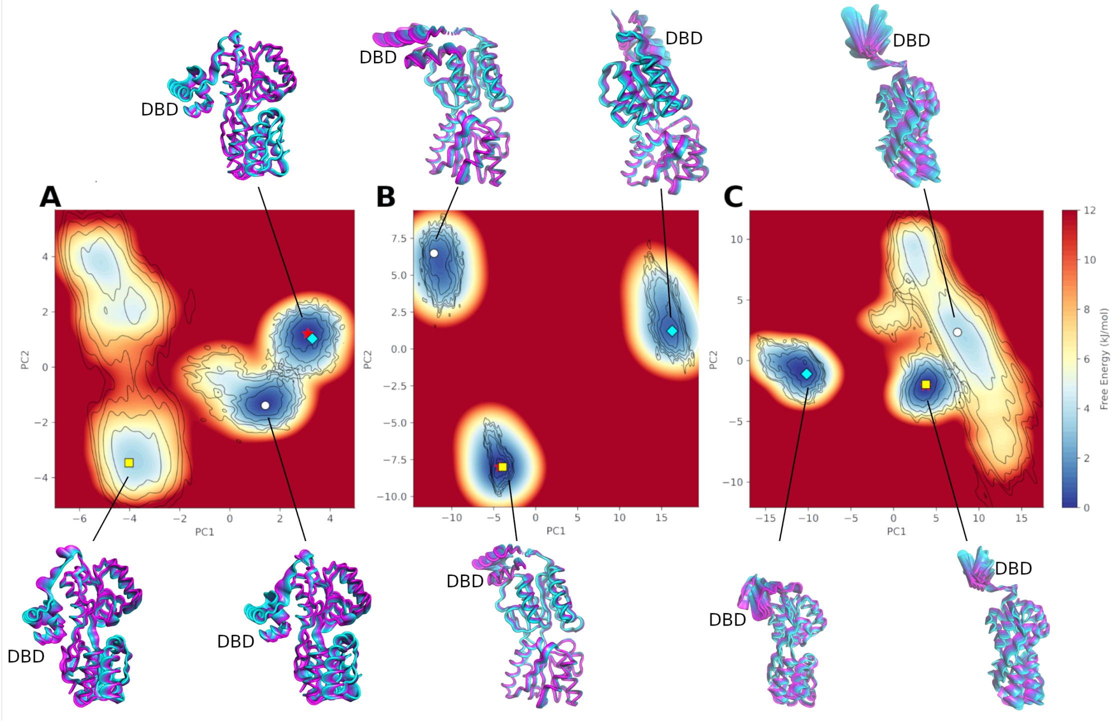


Figure S7: **Free energy landscape (FEL) of the principal motion of Sa-CcpA in the monomeric state.** The FELs of the crystallographic (**A**), extended (**B**), and DNA-bound conformations (**C**) are shown together with the corresponding motion of the DBD in the replica displaying the global minimum energy. Symbols indicate the minimum-energy points for each replica: the star denotes the global minimum, while the circle, diamond, and square correspond to replicas 1, 2, and 3, respectively.

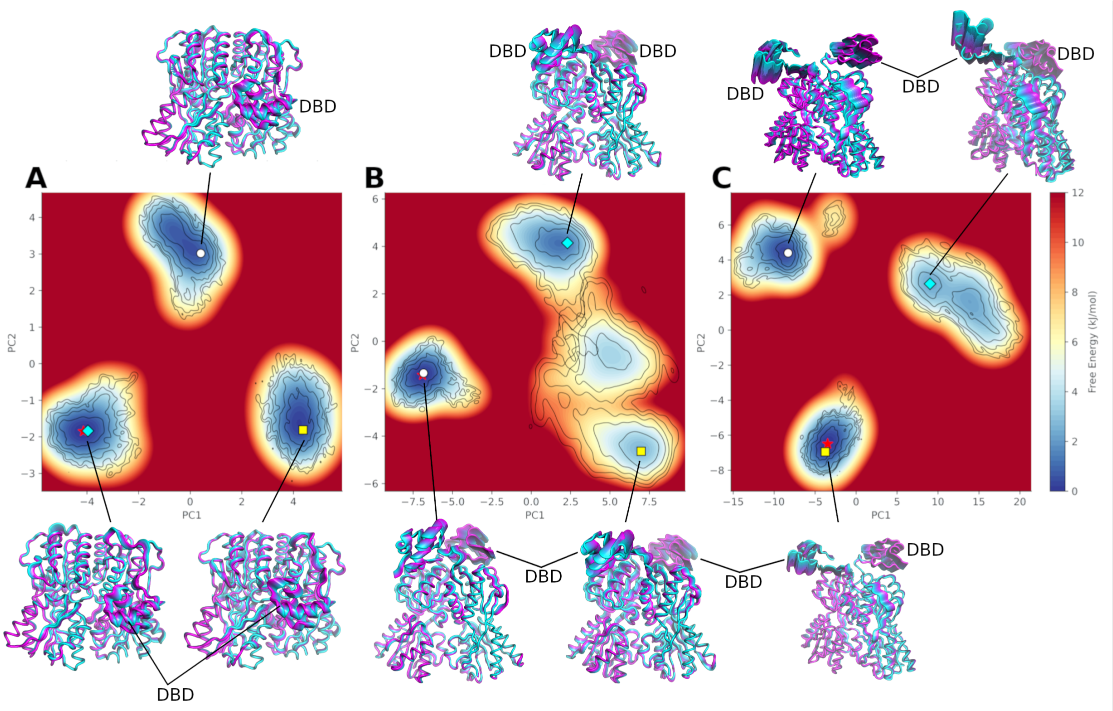


Figure S8: **Free energy landscape (FEL) of the principal motion of Sa-CcpA in the dimeric state.** The FELs of the crystallographic (**A**), extended (**B**), and DNA-bound conformations (**C**) are shown together with the corresponding motion of the DBD in the replica displaying the global minimum energy. Symbols indicate the minimum-energy points for each replica: the star denotes the global minimum, while the circle, diamond, and square correspond to replicas 1, 2, and 3, respectively.

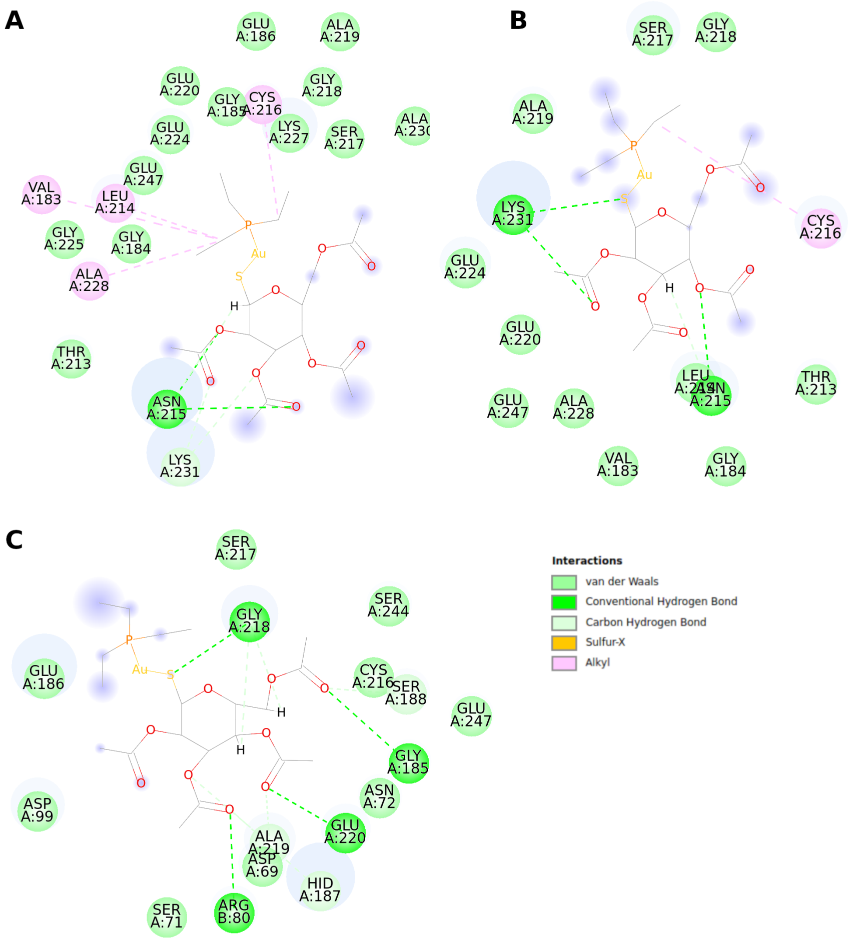


Figure S9: **Molecular interaction between TDP and CcpA obtained from docking simulations.** Interaction maps are shown for three conformational states of the protein: the dimeric form (**A**), the extended DBD (**B**), and the DNA-bound conformation (**C**). The interaction maps were generated using Discovery Studio, with intermolecular contacts colored according to interaction type.

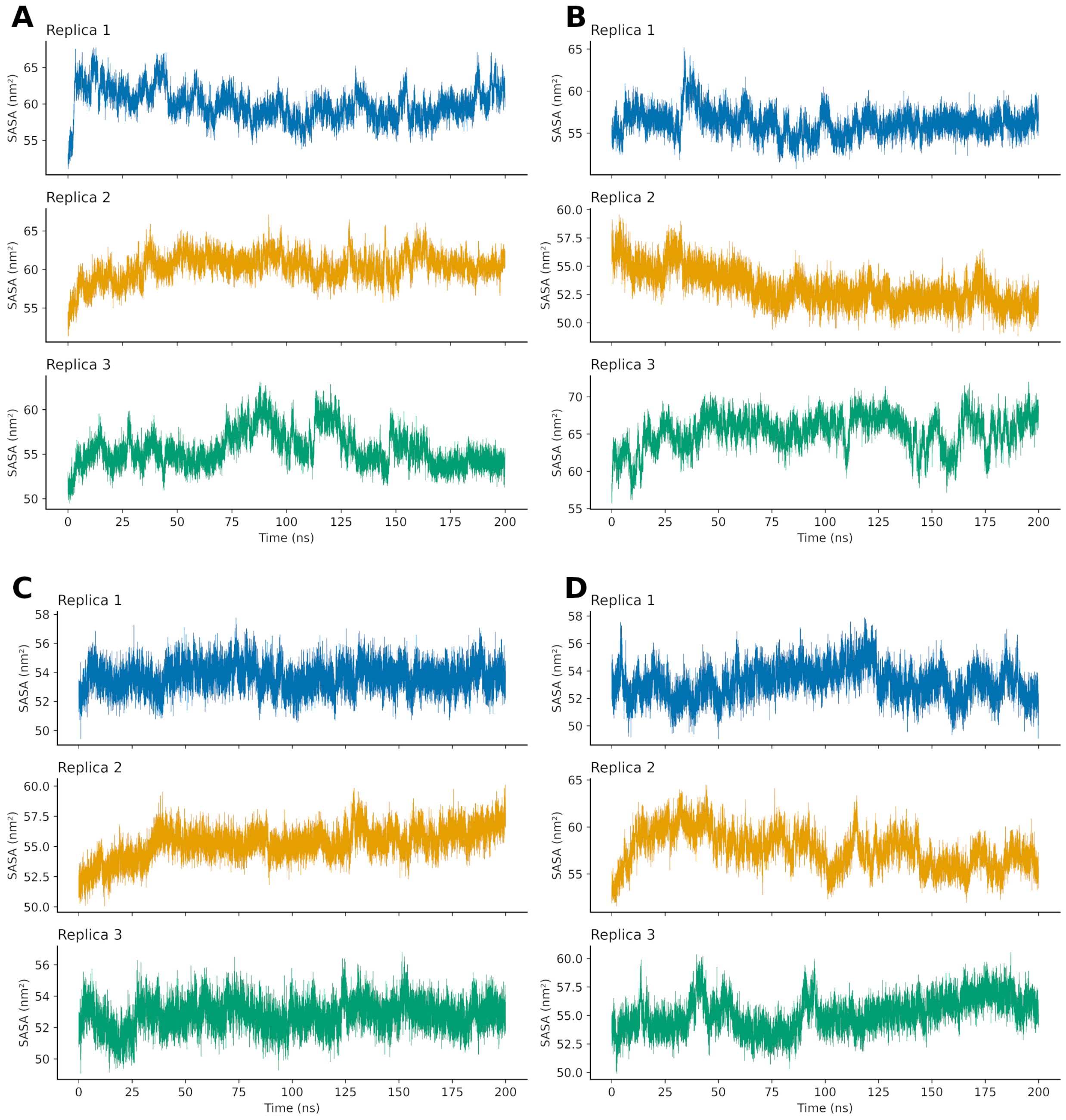


Figure S10: **Solvent accessible surface area of the predicted cavity across independent molecular dynamics simulations**. Each panel shows the SASA calculated along the trajectory for the three independent replicas of each system. (**A**) Monomeric CcpA starting from the crystallographic conformation. (**B**) Monomeric CcpA in the DNA-bound conformation. (**C**) Dimeric CcpA starting from the crystallographic conformation. (**D**) Dimeric CcpA in the DNA-bound conformation.

# Tables

Table S1: **Predicted cavities for Sa-CcpA, Ec-PurR, and Bs-CcpA using CavityPlus.**

| TF | Cavities | Pred Max pKd | Pred Ave pKd | DrugScore | Druggability |
|---|---|---|---|---|---|
| **Sa-ccpA** | 1 | 11.42 | 6.98 | 139.00 | Medium |
| | 2 | 9.96 | 6.03 | -389.00 | Weak |
| | 3 | 8.19 | 5.43 | -1181.00 | Weak |
| | 4 | 8.03 | 5.37 | -1206.00 | Weak |
| | 5 | 6.92 | 4.99 | -875.00 | Weak |
| | 6 | 6.66 | 4.90 | -1227.00 | Weak |
| | 7 | 5.51 | 4.51 | -1523.00 | Weak |
| **Ec-puuR** | 1 | 10.82 | 6.33 | 132.00 | Medium |
| | 2 | 9.38 | 5.83 | 107.00 | Medium |
| | 3 | 8.00 | 5.36 | -775.00 | Weak |
| | 4 | 7.37 | 5.15 | -839.00 | Weak |
| | 5 | 7.18 | 5.08 | -623.00 | Weak |
| | 6 | 5.93 | 4.65 | -1279.00 | Weak |
| **Bs-ccpA** | 1 | 11.13 | 6.98 | 347.00 | Medium |
| | 2 | 10.61 | 6.26 | 260.00 | Medium |
| | 3 | 8.88 | 5.66 | -859.00 | Weak |
| | 4 | 7.83 | 5.30 | -1139.00 | Weak |
| | 5 | 7.30 | 5.12 | -787.00 | Weak |
| | 6 | 7.17 | 5.08 | -927.00 | Weak |
| | 7 | 7.05 | 5.04 | -926.00 | Weak |
| | 8 | 6.40 | 4.81 | -1028.00 | Weak |
| | 9 | 6.30 | 4.78 | -1075.00 | Weak |

Table S2: **Comparison of RMSD values obtained from the structural alignment of Sa-CcpA with the selected set of homologous proteins.** Structural alignments were performed in PyMOL using the functions *align*, *cealign*, and *super*.

| | **Align** | **Cealign** | **Super** |
|---|---|---|---|
| 1b4b | 17.3696098327637 | 17.239688873291 | 15.9135675430298 |
| 1bdh | 1.74480378627777 | 3.82666683197021 | 2.05101418495178 |
| 1bdi | 1.78617978096008 | 4.06880664825439 | 1.87257087230682 |
| 1bib | 20.1898174285889 | 6.10875701904297 | 15.6233615875244 |
| 1cgp | 22.1382617950439 | 5.77211809158325 | 9.05039691925049 |
| 1cma | 21.2823963165283 | 21.3321533203125 | 10.4888324737549 |
| 1cmc | 21.3443698883057 | 20.6451835632324 | 6.59630441665649 |
| 1co0 | 20.980749130249 | 15.6012668609619 | 9.47159099578857 |
| 1efa | 3.61497664451599 | 3.54578185081482 | 3.75258016586304 |
| 1g6n | 21.4795265197754 | 14.1701145172119 | 9.46974086761475 |
| 1h9g | 22.7047138214111 | 10.1908922195435 | 7.89673233032227 |
| 1hw5 | 21.4392528533936 | 6.75303411483765 | 10.9766101837158 |
| 1hxd | 22.4827136993408 | 7.44813108444214 | 1.80551218986511 |
| 1i5z | 21.4485340118408 | 6.68082141876221 | 9.4239501953125 |
| 1i69 | 6.02027225494385 | 5.7119779586792 | 8.81948184967041 |
| 1i6x | 21.3960094451904 | 6.87988758087158 | 9.55783939361572 |
| 1j59 | 21.5665302276611 | 5.7425708770752 | 3.26588106155395 |
| 1jf7 | 1.75962710380554 | 3.82960867881775 | 2.09096932411194 |
| 1jfs | 1.78045189380646 | 3.79195261001587 | 1.98071348667145 |
| 1jh9 | 1.77234923839569 | 3.73967695236206 | 1.85323226451874 |
| 1jhg | 10.532280921936 | 6.86267948150635 | 7.1899676322937 |

Continued on next page

**Table S2 – continued from previous page**

| | Align | Cealign | Super |
|---|---|---|---|
| 1lb2 | 21.53590965271 | 6.10458564758301 | 2.47428297996521 |
| 1lbh | 2.67165923118591 | 2.35329580307007 | 2.76343274116516 |
| 1mj2 | 18.6152095794678 | 10.6720209121704 | 10.6400156021118 |
| 1p4a | 27.6591777801514 | 5.7087984085083 | 22.3575038909912 |
| 1pnr | 1.77147328853607 | 4.07452344894409 | 1.88552033901215 |
| 1qp0 | 1.79729807376862 | 3.87850117683411 | 1.82379579544067 |
| 1qp4 | 1.75548827648163 | 3.79984450340271 | 2.00732469558716 |
| 1qp7 | 1.76717400550842 | 3.79515194892883 | 1.94650149345398 |
| 1qpz | 1.77161943912506 | 4.06311845779419 | 1.94453275203705 |
| 1qqa | 1.75662636756897 | 3.86253070831299 | 1.8523223400116 |
| 1qqb | 1.74830865859985 | 3.79652976989746 | 1.81764912605286 |
| 1rcs | 20.2324028015137 | 7.3280291557312 | 8.53692150115967 |
| 1run | 23.8307971954346 | 6.93965864181519 | 17.611364364624 |
| 1ruo | 22.3662109375 | 5.76438426971436 | 8.83100032806397 |
| 1sxg | 1.51697516441345 | 2.03948020935059 | 1.56731939315796 |
| 1tiq | 15.3483304977417 | 6.04715633392334 | 10.8057146072388 |
| 1tiw | 27.958402633667 | 6.54293346405029 | 16.6070289611816 |
| 1tj0 | 24.8015174865723 | 6.53370189666748 | 16.6095943450928 |
| 1tj1 | 27.943380355835 | 6.55381870269775 | 16.8080005645752 |
| 1to9 | 16.5018939971924 | 23.8978309631348 | 10.4253759384155 |
| 1tro | 19.8695182800293 | 6.99575710296631 | 11.7929077148438 |
| 1trr | 22.5332870483398 | 6.80746459960938 | 12.7956972122192 |
| 1vio | 24.2608108520508 | 30.3188724517822 | 7.850501537323 |
| 1vpw | 1.76738095283508 | 3.82747721672058 | 1.93064594268799 |

Continued on next page

**Table S2 – continued from previous page**

| | Align | Cealign | Super |
|---|---|---|---|
| 1wap | 19.7383499145508 | 6.51950216293335 | 3.68383169174194 |
| 1wet | 1.7820143699646 | 4.07403707504272 | 1.88755857944489 |
| 1wrp | 16.9916801452637 | 6.9201340675354 | 6.75372457504272 |
| 1wrs | 19.5316600799561 | 20.7700805664062 | 11.7309408187866 |
| 1xcb | 24.0001392364502 | 5.98972797393799 | 12.8421468734741 |
| 1xxa | 18.0995635986328 | 7.39816570281982 | 10.4287185668945 |
| 1xxb | 23.0763454437256 | 7.25337791442871 | 11.3708076477051 |
| 1yak | 16.3108177185059 | 6.08150339126587 | 10.3903226852417 |
| 1zay | 1.73206639289856 | 4.07628107070923 | 1.8177387714386 |
| 1zrc | 21.5751247406006 | 6.84727382659912 | 9.42175579071045 |
| 1zrd | 21.5954456329346 | 7.01658487319946 | 9.08209609985352 |
| 1zre | 21.5517196655273 | 6.85002279281616 | 9.0238037109375 |
| 1zrf | 21.5829124450684 | 6.87346410751343 | 3.90359020233154 |
| 1zt9 | 20.9999446868896 | 8.70623016357422 | 11.7657737731934 |
| 2aac | 3.65312552452087 | 7.3985710144043 | 6.38113117218018 |
| 2avx | 14.8990345001221 | 19.9012889862061 | 16.4384269714355 |
| 2b18 | 16.9917297363281 | 6.29469633102417 | 11.5878000259399 |
| 2c96 | 16.1086978912354 | 6.83236122131348 | 13.3332815170288 |
| 2c98 | 16.2273406982422 | 6.65301179885864 | 14.7245292663574 |
| 2c99 | 13.7115602493286 | 6.60686206817627 | 15.1369180679321 |
| 2c9c | 21.1444072723389 | 7.34543466567993 | 15.0555953979492 |
| 2cgp | 21.4830951690674 | 6.98806428909302 | 9.54364967346191 |
| 2dt5 | 18.8847732543945 | 5.89825248718262 | 13.337254524231 |
| 2ewn | 24.1253128051758 | 5.76444101333618 | 6.68475151062012 |

Continued on next page

**Table S2 – continued from previous page**

| | **Align** | **Cealign** | **Super** |
|---|---|---|---|
| 2f78 | 23.2431125640869 | 5.56113576889038 | 4.67005157470703 |
| 2f7a | 22.8837203979492 | 6.4139084815979 | 4.96443605422974 |
| 2f7c | 20.9700527191162 | 6.61490535736084 | 4.61634588241577 |
| 2fzm | 23.602123260498 | 6.61615228652954 | 17.4273300170898 |
| 2fzn | 22.7326755523682 | 6.51635551452637 | 18.1105613708496 |
| 2hza | 21.0460414886475 | 6.89463376998901 | 19.8214797973633 |
| 2jhe | 23.9021987915039 | 31.0477561950684 | 9.97529888153076 |
| 2mbz | 16.5548038482666 | 15.6208992004395 | 9.99174880981445 |
| 2mc0 | 16.1631774902344 | 8.59947299957275 | 5.64919996261597 |
| 2nzu | 1.64317536354065 | 2.35235643386841 | 1.67111301422119 |
| 2okg | 15.1447477340698 | 5.53609275817871 | 16.8620872497559 |
| 2oz9 | 10.5050497055054 | 6.85828590393066 | 7.64284229278564 |
| 2p5m | 20.669771194458 | 10.2653656005859 | 15.7749814987183 |
| 2p9h | 1.59113907814026 | 2.44071316719055 | 1.74756455421448 |
| 2paf | 1.67819726467133 | 2.40671443939209 | 1.83155131340027 |
| 2pe5 | 3.65285658836365 | 3.56073880195618 | 3.80024909973145 |
| 2vii | 16.2306842803955 | 6.80756711959839 | 15.5610122680664 |
| 2vt2 | 23.1326599121094 | 6.64872646331787 | 18.3723621368408 |
| 2vt3 | 20.7714881896973 | 6.34239816665649 | 17.9782199859619 |
| 2zfz | 21.3358707427979 | 12.5047569274902 | 18.9952659606934 |
| 3bni | 15.979058265686 | 27.254861831665 | 4.55405187606812 |
| 3bxe | 15.3433666229248 | 5.19703578948975 | 17.0533847808838 |
| 3bxf | 15.4201908111572 | 5.7489709854126 | 13.9999914169312 |
| 3bxg | 15.3016834259033 | 5.19556570053101 | 16.835563659668 |

Continued on next page

**Table S2 – continued from previous page**

| | Align | Cealign | Super |
|---|---|---|---|
| 3bxh | 15.3000431060791 | 5.15735912322998 | 12.1117334365845 |
| 3cag | 24.0735569000244 | 18.7559127807617 | 17.7628841400146 |
| 3d6y | 20.8832721710205 | 18.8822212219238 | 6.60342645645142 |
| 3d6z | 20.777868270874 | 27.1228828430176 | 6.64589214324951 |
| 3djl | 18.0645637512207 | 6.29737854003906 | 5.276526927948 |
| 3dnt | 20.7842197418213 | 6.51296138763428 | 6.18362712860107 |
| 3e2q | 24.6210441589355 | 6.50864267349243 | 18.0993175506592 |
| 3e2r | 24.5657196044922 | 6.54380464553833 | 13.6900939941406 |
| 3e2s | 24.6103267669678 | 6.80615711212158 | 18.1732158660889 |
| 3edc | 1.62778854370117 | 2.34635210037231 | 1.91855835914612 |
| 3fbr | 20.7881393432617 | 8.28203582763672 | 6.13057422637939 |
| 3fhz | 25.2623653411865 | 24.551441192627 | 15.6176834106445 |
| 3fwr | 23.8170871734619 | 18.1389102935791 | 16.7634601593018 |
| 3fws | 23.7665996551514 | 18.0472946166992 | 16.7672348022461 |
| 3g1l | 15.5991840362549 | 7.41101598739624 | 5.3272762298584 |
| 3g1m | 13.6999835968018 | 7.91714715957642 | 4.84252595901489 |
| 3g1o | 17.5958023071289 | 7.4595103263855 | 4.80142402648926 |
| 3hxu | 16.5164241790771 | 5.97883701324463 | 8.54569721221924 |
| 3hxv | 16.5178833007813 | 5.92713117599487 | 7.77679967880249 |
| 3hxw | 16.513427734375 | 5.91786956787109 | 8.51980781555176 |
| 3hxx | 16.6953830718994 | 5.8687915802002 | 8.47751998901367 |
| 3hxy | 16.6215400695801 | 5.92378902435303 | 8.47554492950439 |
| 3hxz | 16.5681781768799 | 5.96701955795288 | 13.2112159729004 |
| 3hy0 | 16.5522956848145 | 5.95880746841431 | 8.27709102630615 |

Continued on next page

**Table S2 – continued from previous page**

| | Align | Cealign | Super |
|---|---|---|---|
| 3hy1 | 16.64768409729 | 5.23200368881226 | 7.69434547424316 |
| 3hzi | 25.7178783416748 | 6.67579936981201 | 2.53846478462219 |
| 3i54 | 19.3703422546387 | 6.73192119598389 | 16.8187522888184 |
| 3i59 | 24.4145069122314 | 6.49735307693481 | 17.2050628662109 |
| 3ikt | 22.4106521606445 | 6.02620124816895 | 12.7576961517334 |
| 3itg | 22.8415927886963 | 6.51959609985352 | 18.9274253845215 |
| 3kcc | 21.3648548126221 | 6.77622699737549 | 9.34620952606201 |
| 3kos | 10.9661121368408 | 5.9176459312439 | 3.8397421836853 |
| 3laj | 29.8838024139404 | 9.19164276123047 | 21.5199108123779 |
| 3lap | 30.0211048126221 | 35.6938285827637 | 20.122579574585 |
| 3mzh | 22.2444362640381 | 6.75082540512085 | 17.4958057403564 |
| 3n4m | 21.5833492279053 | 6.54758358001709 | 9.06173896789551 |
| 3o8g | 17.6004581451416 | 7.45235443115234 | 4.89457273483276 |
| 3o8h | 13.6801862716675 | 7.41772651672363 | 4.75972890853882 |
| 3od2 | 27.3942642211914 | 7.22226428985596 | 22.1825313568115 |
| 3q0u | 17.512414932251 | 7.41700983047485 | 4.8452672958374 |
| 3q0v | 18.8404693603516 | 7.4207034111023 | 4.72311925888062 |
| 3q0w | 15.6552953720093 | 7.42626142501831 | 4.78066921234131 |
| 3q1m | 24.4120254516602 | 23.1165466308594 | 6.40150785446167 |
| 3q3s | 13.7098150253296 | 7.42704916000366 | 4.80911016464233 |
| 3q5p | 24.3004684448242 | 12.7160177230835 | 6.37445402145386 |
| 3q5r | 24.3586597442627 | 11.6383142471313 | 6.32687139511108 |
| 3q5s | 24.2687397003174 | 12.8702421188354 | 6.44461488723755 |
| 3qf3 | 22.8375759124756 | 7.50680351257324 | 3.43299651145935 |

Continued on next page

**Table S2 – continued from previous page**

| | Align | Cealign | Super |
|---|---|---|---|
| 3r61 | 22.2030220031738 | 7.52633953094482 | 7.06809616088867 |
| 3rdi | 21.3867530822754 | 9.67756366729736 | 9.42597007751465 |
| 3rou | 21.4128074645996 | 6.67534446716309 | 5.48214912414551 |
| 3rpq | 21.5984706878662 | 6.88768339157105 | 9.59899997711182 |
| 3ryp | 21.4733791351318 | 6.8757791519165 | 8.75064373016357 |
| 3ryr | 21.6005268096924 | 6.8632230758667 | 9.40248489379883 |
| 3sdg | 14.9459476470947 | 7.42567110061646 | 4.84347009658814 |
| 3sfi | 14.5169458389282 | 6.42254781723022 | 4.85996961593628 |
| 3tb6 | 1.75755715370178 | 2.60091853141785 | 1.98496556282043 |
| 3tp0 | 13.9206838607788 | 7.43976926803589 | 4.84119749069214 |
| 3tpt | 20.8124809265137 | 6.50265455245972 | 6.97565317153931 |
| 3tpv | 21.7587242126465 | 6.6545467376709 | 5.46872568130493 |
| 3u33 | 18.1215782165527 | 6.29720401763916 | 8.52483654022217 |
| 3whb | 24.4783382415771 | 22.6763610839844 | 6.93638706207275 |
| 3whc | 24.5766010284424 | 16.096715927124 | 7.75732421875 |
| 4a2u | 24.6725807189941 | 6.55902242660522 | 16.9771308898926 |
| 4aci | 23.2875957489014 | 8.99062919616699 | 4.54298782348633 |
| 4af5 | 17.4729480743408 | 7.87662601470947 | 9.03423309326172 |
| 4b27 | 17.6047897338867 | 5.86824178695679 | 3.69193935394287 |
| 4dw6 | 13.8221950531006 | 7.40133333206177 | 4.71982955932617 |
| 4esq | 17.3111171722412 | 20.1832714080811 | 13.5355501174927 |
| 4fe7 | 17.7791900634766 | 4.74853849411011 | 4.32107877731323 |
| 4ft8 | 21.4777965545654 | 6.7055778503418 | 11.1404399871826 |
| 4go1 | 18.7732810974121 | 6.65041399002075 | 11.4914855957031 |

Continued on next page

**Table S2 – continued from previous page**

| | Align | Cealign | Super |
|---|---|---|---|
| 4i01 | 21.4225521087646 | 5.80406188964844 | 9.73109149932861 |
| 4i02 | 16.6767024993896 | 6.81882333755493 | 9.4763765335083 |
| 4i09 | 21.4612331390381 | 6.67270135879517 | 11.1238813400269 |
| 4i0a | 21.4803714752197 | 6.64441823959351 | 8.83985710144043 |
| 4i0b | 21.7976989746094 | 6.8724102973938 | 9.12994289398193 |
| 4jny | 24.7372341156006 | 6.54783248901367 | 18.014762878418 |
| 4jnz | 24.3139972686768 | 6.55712938308716 | 13.7895355224609 |
| 4l4z | 19.5231895446777 | 6.65481042861939 | 8.99317646026611 |
| 4l50 | 19.4969348907471 | 5.93576526641846 | 9.18466567993164 |
| 4l51 | 19.3774299621582 | 6.08936882019043 | 12.7573261260986 |
| 4l5j | 19.4114837646484 | 6.10086679458618 | 18.827205657959 |
| 4lj3 | 25.6281719207764 | 6.44508504867554 | 16.2167167663574 |
| 4lq2 | 21.0585861206055 | 5.88810729980469 | 3.54168081283569 |
| 4lq5 | 21.0876617431641 | 5.60427236557007 | 3.05589723587036 |
| 4lrz | 16.1605758666992 | 15.953932762146 | 8.74159240722656 |
| 4lyk | 16.2172183990479 | 6.35920429229736 | 11.0271501541138 |
| 4m3b | 14.9501399993896 | 7.40777826309204 | 4.7619571685791 |
| 4m3d | 14.5907926559448 | 7.42437887191772 | 5.15676927566528 |
| 4m3e | 15.7130107879639 | 7.42829704284668 | 4.9198579788208 |
| 4m3g | 20.6815929412842 | 7.41302490234375 | 4.91191864013672 |
| 4nb5 | 22.0286540985107 | 28.4683990478516 | 11.7765207290649 |
| 4o8a | 25.0071334838867 | 6.51913261413574 | 13.8844976425171 |
| 4op0 | 16.8905048370361 | 22.2943782806396 | 6.33110570907593 |
| 4oqq | 20.0474987030029 | 5.0105767250061 | 7.29681158065796 |

Continued on next page

**Table S2 – continued from previous page**

| | Align | Cealign | Super |
|---|---|---|---|
| 4qnm | 19.7576522827148 | 7.03897047042847 | 14.9988794326782 |
| 4qnr | 17.2032737731934 | 6.58211278915405 | 14.8649225234985 |
| 4qos | 19.7278938293457 | 7.19034147262573 | 13.4618110656738 |
| 4r3l | 16.4647369384766 | 5.91740703582764 | 12.5103549957275 |
| 4r4e | 19.5687351226807 | 12.2397327423096 | 5.63931751251221 |
| 4r8h | 21.5581016540527 | 6.90347671508789 | 9.63866996765137 |
| 4rzt | 1.69404006004334 | 2.44437098503113 | 1.92122137546539 |
| 4u0v | 27.8587207794189 | 5.21490812301636 | 4.13492155075073 |
| 4u0w | 27.886589050293 | 4.93877935409546 | 4.15868043899536 |
| 4w97 | 16.8555870056152 | 12.4059858322144 | 9.01523113250732 |
| 4wf2 | 21.2865447998047 | 8.68309879302979 | 3.0887930393219 |
| 4xtu | 17.23610496521 | 13.1335039138794 | 5.35876798629761 |
| 4xtv | 17.2467136383057 | 13.1853361129761 | 6.38651275634766 |
| 4xtw | 17.2446537017822 | 13.1247568130493 | 6.40641117095947 |
| 4xtx | 17.2419605255127 | 13.172758102417 | 6.47858428955078 |
| 4xty | 17.2311916351318 | 13.134069442749 | 5.4340877532959 |
| 4xtz | 17.2612476348877 | 18.620979309082 | 6.45663404464722 |
| 4xu0 | 17.2522659301758 | 18.6068878173828 | 6.42800664901733 |
| 4xu1 | 17.2252941131592 | 8.26254749298096 | 6.43021678924561 |
| 4xu2 | 21.1700382232666 | 13.1576633453369 | 6.45497703552246 |
| 4xu3 | 21.1287879943848 | 13.3279714584351 | 6.42189502716064 |
| 4xxh | 2.31363749504089 | 2.78422093391418 | 2.2159264087677 |
| 4zsk | 16.8955345153809 | 5.71671867370606 | 3.00340008735657 |

Table S3: **Results obtained from the molecular docking of the inhibitors Auronafin and TDP across the different conformations of Sa-CcpA.**

| Systems | Ligand | Score | S (PLP) | S (hbond) | S (metal) | DE (clash) | DE (tors) |
|---|---|---|---|---|---|---|---|
| Crystal | Auronafin | -154.11 | 154.99 | 1.65 | 0.00 | 0.12 | 3.03 |
| | TDP | -37.31 | 36.72 | 0.00 | 0.00 | 0.00 | 0.55 |
| monomer_1 | Auronafin | 39.68 | -36.13 | 1.35 | 0.00 | 0.00 | 0.79 |
| | TDP | 40.15 | -34.42 | 1.97 | 0.00 | 0.00 | 0.34 |
| monomer_2 | Auronafin | 50.24 | -47.31 | 1.66 | 0.00 | 0.00 | 1.59 |
| | TDP | 54.01 | -51.24 | 1.02 | 0.00 | 0.00 | 0.40 |
| monomer_ext1 | Auronafin | 50.31 | -45.07 | 2.00 | 0.00 | 0.00 | 0.95 |
| | TDP | 50.55 | -44.99 | 2.00 | 0.00 | 0.00 | 0.48 |
| monomer_ext2 | Auronafin | 54.32 | -49.67 | 2.00 | 0.00 | 0.00 | 1.27 |
| | TDP | 41.29 | -36.16 | 1.98 | 0.00 | 0.00 | 0.67 |
| monomer_DNA1 | Auronafin | -77.64 | 85.96 | 4.10 | 0.00 | 0.00 | 2.95 |
| | TDP | 21.77 | -31.34 | 1.45 | 0.00 | 13.44 | 0.50 |
| monomer_DNA2 | Auronafin | 55.13 | -52.66 | 1.00 | 0.00 | 0.00 | 0.92 |
| | TDP | 42.90 | -40.02 | 1.00 | 0.00 | 0.00 | 0.31 |
| dimer_1 | Auronafin | -98.84 | 105.35 | 3.00 | 0.00 | 0.00 | 2.30 |
| | TDP | 37.09 | -34.90 | 0.82 | 0.00 | 0.00 | 0.39 |
| dimer_2 | Auronafin | 38.37 | -38.52 | 0.00 | 0.00 | 0.00 | 0.66 |
| | TDP | 42.49 | -41.03 | 0.69 | 0.00 | 0.00 | 0.56 |
| dimer_ext1 | Auronafin | 64.71 | -61.05 | 2.17 | 0.00 | 0.00 | 2.27 |
| | TDP | 53.41 | -47.81 | 1.94 | 0.00 | 0.00 | 0.36 |
| dimer_ext2 | Auronafin | -162.42 | 158.90 | 0.00 | 0.00 | 0.17 | 2.62 |
| | TDP | -25.59 | 30.90 | 1.97 | 0.00 | 0.00 | 0.55 |
| dimer_DNA1 | Auronafin | 49.66 | -45.35 | 1.98 | 0.00 | 0.00 | 1.38 |
| | TDP | 55.90 | -51.47 | 1.48 | 0.00 | 0.00 | 0.26 |
| dimer_DNA2 | Auronafin | 47.54 | -46.67 | 1.00 | 0.00 | 0.00 | 1.67 |
| | TDP | 46.76 | -43.79 | 1.00 | 0.00 | 0.00 | 0.27 |
